# Hybrid Integration of InGaN Lasers in a Foundry‐Fabricated Visible‐Light Photonics Platform

**DOI:** 10.1002/nap2.70037

**Published:** 2026-04-10

**Authors:** Xin Mu, Frank Weiss, Hongyao Chua, Robert Lawrowski, Jared C. Mikkelsen, John N. Straguzzi, Hannes Wahn, Piyush Kumar, Guo‐Qiang Lo, Joyce K. S. Poon, Mariel Jama, Wesley D. Sacher

**Affiliations:** ^1^ Max Planck Institute of Microstructure Physics Halle Germany; ^2^ University of Toronto Toronto Ontario Canada; ^3^ Advanced Micro Foundry Pte. Ltd. Singapore; ^4^ ams OSRAM International GmbH Regensburg Germany

**Keywords:** flip‐chip bonding, laser integration, silicon photonics, visible‐light integrated photonics

## Abstract

Visible‐spectrum photonic integrated circuits (PICs) present compact, scalable solutions for quantum computing, biosensing, and virtual/augmented reality. Realizing their potential requires scalable visible‐light‐source integration methods compatible with high‐volume manufacturing and efficient optical coupling. Here, we demonstrate passive‐alignment flip‐chip bonding of 450‐nm InGaN laser diodes onto a foundry‐fabricated visible‐light silicon (Si) photonics platform with silicon nitride (SiN) waveguides, thermo‐optic devices, and photodetectors. Hybrid laser integration is realized using a sub‐micron‐precision die bonder with a vision alignment system and heatable pickup tool, allowing placement of multiple lasers on a single Si chip. Co‐design of the lasers and Si photonics, with lithographically defined alignment marks and mechanical stoppers, enables precise post‐bonding alignment. Efficient coupling to SiN waveguides is demonstrated, with a 1.1 dB minimum coupling loss. We measure 19.0 mW mean on‐chip optical power at 90 mA drive current; at 185 mA, maximum on‐chip power reaches 60.7 mW, the highest reported for hybrid‐integrated visible‐spectrum lasers, to our knowledge. A maximum wall‐plug efficiency of 7.8% is measured. An active PIC is shown, integrating a bonded laser, photodetector power monitor, and thermo‐optic switch for routing and variable attenuation. This work highlights passive‐alignment flip‐chip bonding as a practical, high‐performance approach for integrating lasers onto visible‐spectrum PICs.

## Graphical abstract

We demonstrate scalable visible‐light‐source integration via passive‐alignment flip‐chip bonding of 450‐nm InGaN lasers onto foundry‐fabricated SiN‐on‐SOI photonics. A sub‐micron‐precision die bonder places multiple lasers per chip, while lithographically defined alignment marks and mechanical stoppers ensure accurate post‐bonding alignment. Coupling loss is as low as 1.1 dB, delivering up to 60.7 mW on‐chip power and 7.8% wall‐plug efficiency.

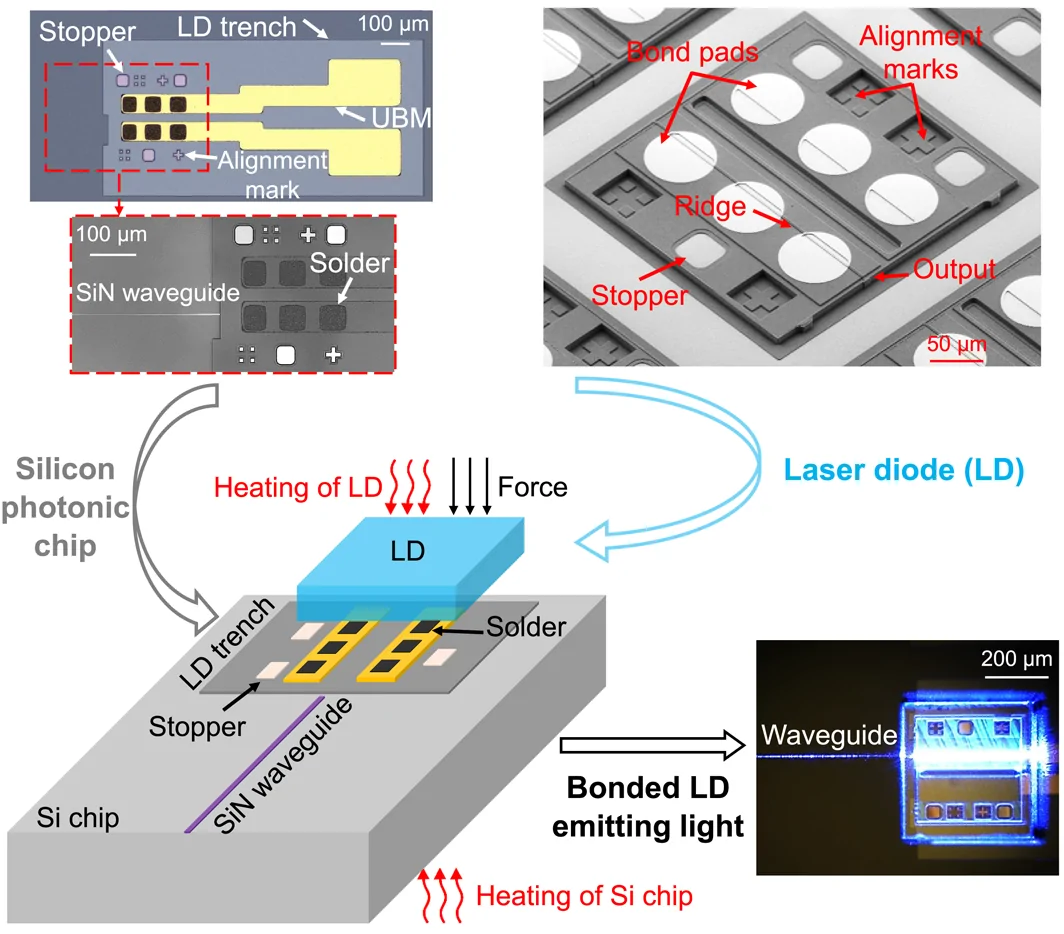

## 1 Introduction

Silicon (Si) photonics in the visible spectrum represents a new frontier for photonic integrated circuits (PICs), broadening the range of applications beyond that of conventional Si photonics in the telecommunications O‐ and C‐bands [1, 2, 3, 4]. Extending Si photonics into the visible spectrum unlocks opportunities in emerging technologies, including PIC‐based microdisplays for virtual/augmented reality [5, 6, 7], quantum computing [8, 9], biosensing [10], neurotechnology [11, 12, 13], as well as free space and underwater optical communications [14, 15]. To date, substantial progress has been made in establishing the fundamental on‐chip components, such as passive silicon nitride (SiN) and aluminum oxide waveguide devices [2, 16, 17], beam scanners [18, 19], optical switches and modulators [20, 21, 22, 23], and photodetectors [24, 25, 26]. Despite these advances, realizing the full potential of visible‐light Si photonics will require integrated light sources that collectively span the visible spectrum.

A key challenge in integrating lasers onto Si photonic platforms is the poor light‐generation efficiency of the standard waveguide materials: Si has an indirect bandgap, and the SiN used here is an amorphous, wide‐bandgap dielectric. Integration with direct‐bandgap semiconductors is therefore essential for efficient on‐chip light sources. Various laser integration strategies have been investigated, including wafer bonding [27], direct epitaxial growth [28], micro‐transfer printing [29], and flip‐chip bonding [30]. With most prior work focused on telecommunications wavelengths using indium phosphide gain media, visible‐spectrum integration requires multiple gain materials, such as indium gallium nitride (InGaN) for blue/green lasers and gallium arsenide–based compounds for red lasers, along with wavelength‐specific optimization of device compositions. Hybrid integration of pre‐fabricated laser diodes (LDs) offers a practical route to incorporating visible light sources into Si photonics, accommodating the diverse materials and the wide wavelength range of the visible spectrum. Recent work has explored actively aligning edge‐emitting LDs to Si photonic chip facets via butt coupling [31, 32], achieving precise alignment and high coupling efficiency but relying on slow, feedback‐based assembly and limiting integration density due to facet and LD submount size constraints. These factors bottleneck assembly throughput for volume production and restrict PIC complexity.

An alternative to active‐alignment techniques for hybrid laser integration is passive‐alignment flip‐chip bonding, a method that has been widely demonstrated in conventional infrared Si photonics [33, 34, 35]. Here, etched recesses (LD trenches or bonding sockets) are defined on the Si photonic platform. Lithographically defined alignment marks on both the LDs and Si chips enable precise in‐plane LD positioning, while mechanical stoppers on both dies, often formed using etch‐stop layers, ensure accurate vertical self‐alignment via contact between corresponding features. Electrical connection is achieved by interfacing the LD metal contacts with solder bumps and under‐bump metallization (UBM) pads on the Si chips. Sub‐micron post‐bonding alignment of LDs is typically achieved using commercial die bonders. These systems are widely used in microelectronics packaging, and existing models are capable of high‐throughput assembly. By combining precisely defined on‐chip alignment features with advanced die‐bonding tools, passive‐alignment flip‐chip bonding offers a scalable route for hybrid laser integration, enabling high coupling efficiency, high‐throughput manufacturing, and compatibility with multiple laser types. It also supports pre‐screening and burn‐in testing of LDs to identify known‐good dies, reducing the impact of LD yield on packaged PIC yield. Although proven in infrared Si photonics, applying this approach to visible‐light platforms is more challenging due to the smaller optical mode size of LDs at shorter wavelengths, which tightens alignment tolerances. Consistent with this, recent demonstrations of visible‐spectrum LDs flip‐chip bonded onto PICs have thus far shown limited coupled optical powers. Reference [36] reported a PIC‐based external cavity laser with a flip‐chip bonded red gain chip, although lasing was not achieved in the prototype. Reference [7] demonstrated red, green, and blue flip‐chip bonded lasers on a SiN PIC, with a combined maximum output power of 10 mW.

In this work, we demonstrate the hybrid integration of 450‐nm InGaN laser diodes into a foundry‐fabricated visible‐light Si photonics platform via passive‐alignment flip‐chip bonding, using localized heating compatible with bonding multiple LDs per Si chip. The platform integrates SiN nanophotonic waveguides, thermo‐optic tuners, and waveguide‐coupled Si photodetectors [3, 37]. From 40 bonded samples, we achieve a maximum on‐chip optical power of 60.7 mW and wall‐plug efficiency (referenced to on‐chip power) of up to 7.8%. To the best of our knowledge, this power is a record among visible‐spectrum hybrid‐integrated lasers. The devices maintain stable operation at temperatures up to 80°C, and statistical analysis confirms sub‐micron post‐bonding alignment within the placement accuracy of our die bonder. Although this accuracy constrains assembly yield, we outline pathways for improvement. We also present a PIC with a flip‐chip bonded LD, a monolithically integrated photodetector, and a thermo‐optic switch, demonstrating the platform's core active functions. Overall, this work establishes a scalable platform for laser integration in visible‐light Si photonics, paving the way for advanced active PICs with arrays of hybrid‐integrated visible lasers. Leveraging foundry fabrication and high‐throughput flip‐chip assembly, the approach is poised to scale in complexity and manufacturing volume, enabling PIC‐based solutions for emerging visible‐spectrum applications. An initial report of these results was presented in our conference abstract, Ref. [38].

## 2 Design and Fabrication

Figure 1a illustrates the schematic of the visible‐light Si photonics platform, featuring SiN waveguides, thermo‐optic switches, waveguide‐coupled photodetectors, and structures for hybrid integration of laser diodes. The platform was fabricated at Advanced Micro Foundry on 200‐mm diameter silicon‐on‐insulator (SOI) wafers [39]. The fabrication process began with the formation of 220‐nm‐thick Si photodetectors, including Si patterning and doping steps. Subsequently, SiN waveguides were defined via plasma‐enhanced chemical vapor deposition, 193‐nm deep ultraviolet photolithography, and reactive ion etching. Full‐ and partial‐etch steps were used for definition of 120‐ and 60‐nm‐thick waveguides. Each Si PIN‐junction photodetector was located beneath an SiN waveguide, allowing evanescent coupling of guided light from the SiN waveguide into the underlying Si photodetector. Next, metallization steps were performed with two aluminum routing layers and interlayer vias, as well as the titanium nitride (TiN) resistive heaters. Chemical mechanical polishing was used for layer planarization. ${\text{SiO}}_{2}$ and Si etch steps defined the flip‐chip bonding sockets (LD trenches), mechanical stoppers (for vertical positioning), and alignment marks (for in‐plane alignment), Figure 1b. The Si device layer of the SOI wafers served as both the top surface of the mechanical stoppers and as an etch stop during stopper patterning, thereby minimizing height variability and enabling precise vertical alignment between the stoppers and the waveguides. Under‐bump metallization and gold–tin (AuSn) solder bumps were patterned within the bonding sockets to interface with the metal contacts (bond pads) of LDs for electrical connectivity. Two types of solder bump patterns were implemented—rows of discrete bumps in Figure 1b and continuous stripes in Section 5. The LD trench configuration enables direct thermal contact between the LD and the Si substrate through thin solder and UBM layers, enabling efficient heat sinking. The fabrication process concluded with etching of deep Si trenches to form the chip facets, followed by wafer dicing.

**FIGURE 1:**
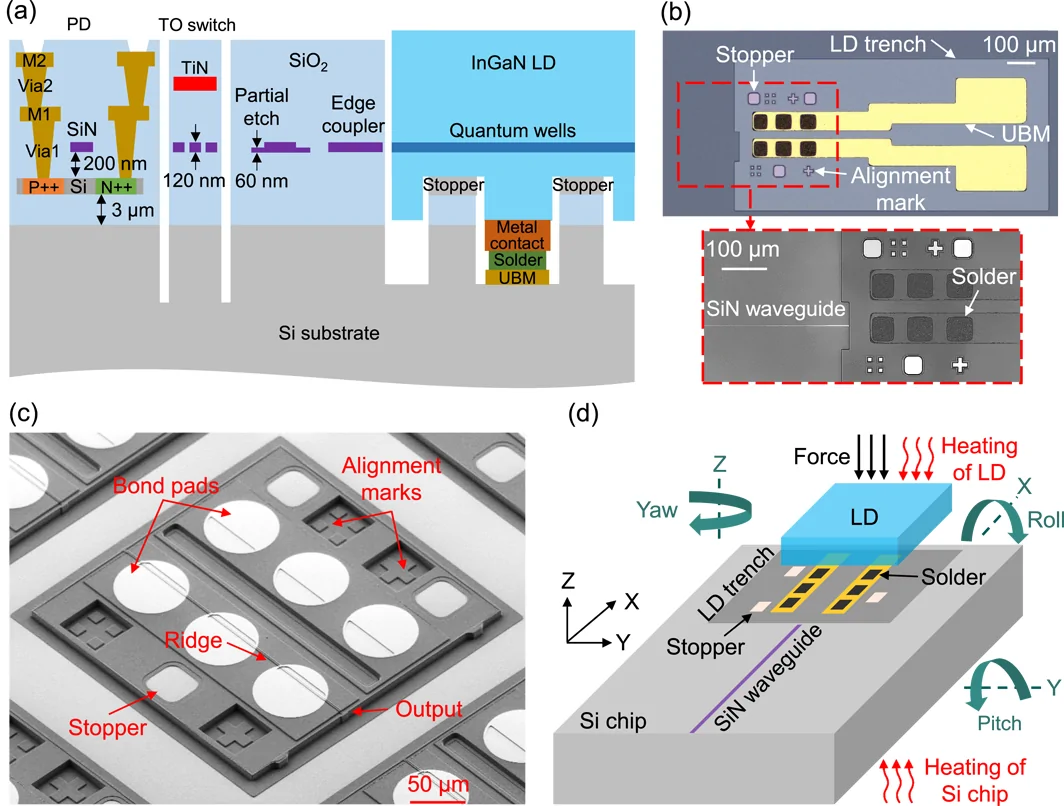
Hybrid integration of InGaN laser diodes (LDs) in a visible‐light Si photonics platform. (a) Cross‐section of the photonics platform. (b) (Top) Optical micrograph of an LD bonding socket on a Si photonic chip and (bottom) confocal laser scanning micrograph of the socket with improved visibility of the SiN waveguide. (c) Scanning electron micrograph of the top surface of an InGaN LD before singulation. (d) Conceptual illustration of the flip‐chip LD bonding method, with an LD close to the socket. The definition of the coordinate system and rotation axes is given. M, metal; PD, photodetector; SiN, silicon nitride; TiN, titanium nitride; TO switch, thermo‐optic switch; UBM, under‐bump metallization.

In parallel, blue (≈450 nm) edge‐emitting, single‐spatial‐mode, InGaN laser diodes were fabricated by ams OSRAM [40]. Each laser diode chip was ≈100 μm thick with a footprint of ≈400 μm × 400 μm, Figure 1c. The InGaN LDs were co‐designed in conjunction with the bonding sockets on the Si photonic platform, specifically for hybrid laser integration with passive‐alignment flip‐chip bonding. Mechanical stopper trenches and alignment marks were formed on the LDs by trench etching, matching counterparts on the Si photonic chips. The LDs featured top‐side P and N metal contacts, allowing for electrical interfacing with the pre‐patterned solder bumps and UBM pads on the Si chips. Precise lithography and etching of the LDs and Si chips ensured that the alignment marks and stoppers provided accurate in‐plane and vertical positioning during placement. SiN edge couplers, located at the facets of the bonding sockets on the Si photonic chips, coupled the emitted light from the flip‐chip bonded lasers into the SiN waveguides (Figure 1d). The detailed laser‐to‐waveguide coupler design is discussed in Section 3.2.

## 3 Hybrid Integration Method

### 3.1 Flip‐Chip Bonding Process

Hybrid laser integration via passive‐alignment flip‐chip bonding was implemented using a semi‐automated die bonder with sub‐micron placement accuracy (Fineplacer sigma, Finetech), Figure 2a. The system picked up the laser diode using a vacuum pickup tool and performed an aligned touchdown onto a bonding socket on the Si photonic chip, applying a controlled sequence of pressure and heat for thermocompression bonding, as conceptually illustrated in Figure 1d. The die bonder featured a vision alignment module that overlaid live microscope images of the laser diode (held by the vacuum pickup tool) and the Si photonic chip (mounted on the translation stage of the die bonder), enabling visualization of the relative positions of cross‐shaped alignment marks on both chips (inset, Figure 2a). A side‐view microscope facilitated real‐time monitoring of the LD touchdown onto the Si photonic chip. Figure 2b shows the side view of the LD approaching the Si photonic chip, taken with a microscope different from the side‐view microscope of the die bonder. The heatable vacuum pickup tool (Figure 2c) allowed localized heating of the LD to selectively melt solder bumps only within the targeted bonding socket. This selective heating enabled sequential integration of multiple LDs onto a single Si chip (Figure 2d). Supplemental heat was applied to the Si photonic chip through the die bonder alignment stage, reducing the temperature required by the pickup tool to reach the solder melting point, which in turn minimized thermal exposure to the LD.

**FIGURE 2:**
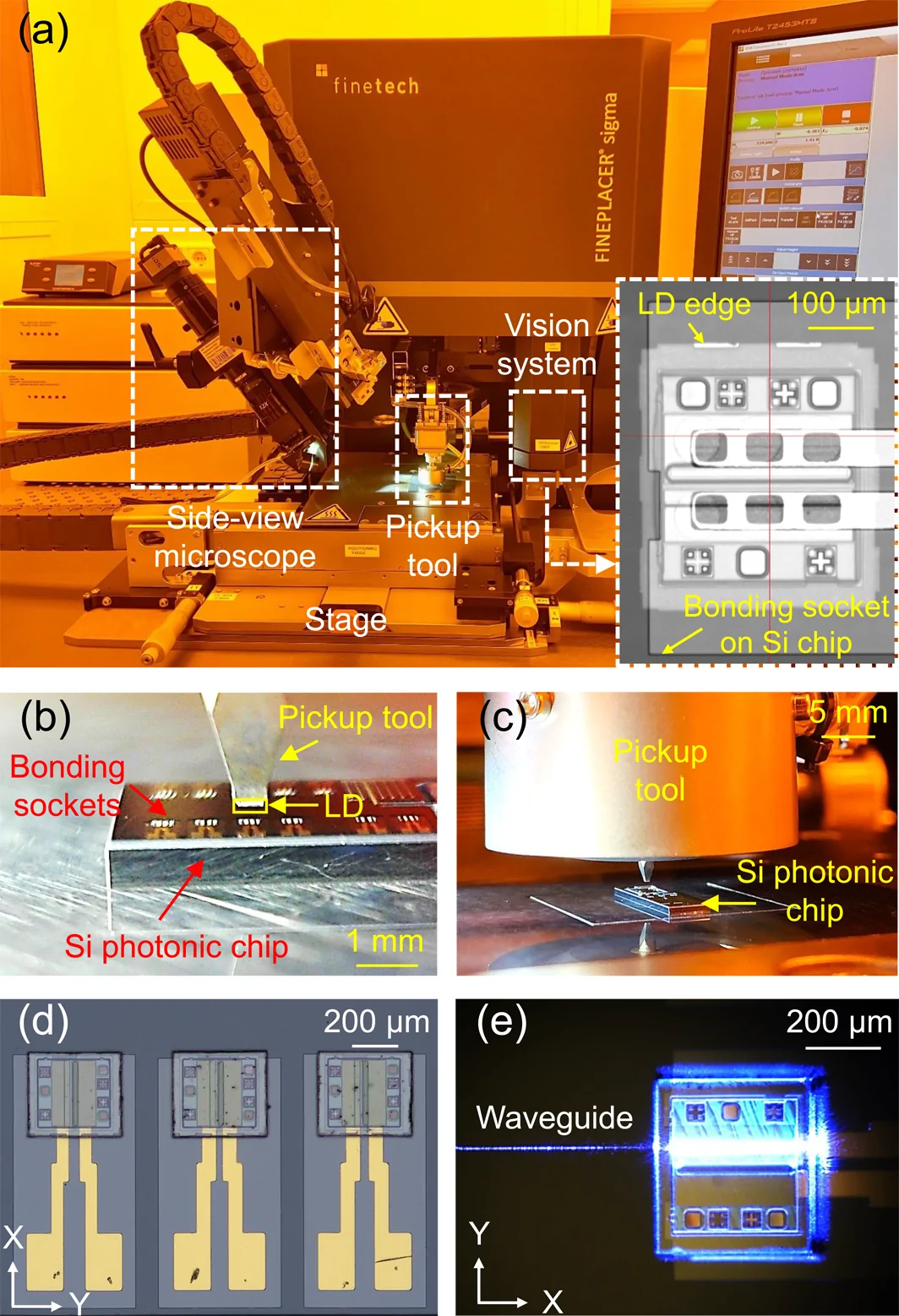
Flip‐chip bonding technique. (a) Photograph of the die bonder with a vision alignment system, a side‐view microscope, and a heatable pickup tool. Inset: overlaid view from the vision system of an LD and a bonding socket prior to bonding. (b) Micrograph of an LD held by the pickup tool approaching a Si photonic chip; the image was captured with a second side‐view microscope not shown in (a). (c) Photograph of the pickup tool above a Si photonic chip. (d) Micrograph of three flip‐chip bonded LDs on one Si photonic chip. (e) Micrograph of a bonded LD with emitted light coupled to a SiN waveguide.

The detailed flip‐chip bonding process began with securing the Si photonic chip onto the translation stage of the die bonder using vacuum suction. Next, an InGaN laser diode, stored with the contact surface down in a nano‐device tray (NDT‐XT‐RA, Gel‐Pak), was picked up by the heatable vacuum pickup tool. Both the Si photonic chip and the LD were then pre‐heated to 270°C, a temperature below the melting point of the AuSn solder. This step minimized the temperature difference between the two chips in the subsequent bonding process, during which the pickup tool was heated up to 400°C, thereby reducing alignment offsets caused by thermal expansion. The target bonding socket on the Si photonic chip was positioned beneath the LD using the translation stage. Precise alignment was achieved by manually centering the alignment marks on both the LD and the Si photonic chip using the vision system. Once aligned, the LD was lowered onto the bonding site, and the thermal bonding profile was initiated upon detecting a touchdown force of 2 N. During the bonding process, the Si chip temperature was maintained at 270°C, while the pickup tool was heated to a peak temperature of 400°C at a ramp rate of 5°C/s. After heating at 400°C for 30 s, both the translation stage and the pickup tool were cooled to 40°C at a rate of 5°C/s using ambient air delivered through vent holes in the stage. A constant touchdown force of 2 N was maintained throughout the process to secure the LD in position and ensure consistent mechanical contact with the bonding socket on the Si photonic chip. Following cooling and solidification of the solder bumps, vacuum suction from the pickup tool was released, and the tool was retracted.

Because of the tapered profile and small tip of the pickup tool as well as the contact with the lower‐temperature Si chip, the LD temperature during bonding was expected to remain below the 400°C tool setting. The peak temperature was estimated at ≈315°C between the pickup tool (400°C) and the stage (270°C), measured using a thermometer (Voltcraft, 302K/J) with a thermocouple (of similar dimensions to the LD) placed between them. These measurements, combined with iterative adjustment of bonding parameters including force, ramp rate, and duration, informed process development aimed at achieving consistent post‐bonding placement accuracy and solder joint integrity. A complete flip‐chip bonding cycle, including sample loading, LD pickup, alignment, thermocompression bonding, and cooling, required ≈15 min. Figure 2e shows a hybrid‐integrated LD following the flip‐chip bonding procedure, with blue emitted light coupled into a SiN photonic waveguide via an edge coupler. Detailed characterization of the LDs is presented in Section 4.1.

### 3.2 Simulated Alignment Sensitivity

To enable efficient optical coupling between the flip‐chip‐bonded laser diodes and Si photonic chips, two types of LD‐to‐waveguide couplers were evaluated. The first design, a SiN inverse taper coupler, begins with a 150‐nm‐width tip at the bonding socket facet, expanding to a 400‐nm‐wide single‐mode waveguide over a 150‐μm length. The second design, a SiN taper coupler, narrows from a 3‐μm width at the socket facet to 400 nm over a length of 190 μm. The inverse taper is implemented in full thickness (120 nm) SiN, whereas the taper coupler uses the partially etched 60‐nm‐thick SiN layer. Figure 3a presents simulated optical mode profiles at the LD emission facet and the coupler facets, obtained using a finite‐difference eigenmode solver (Ansys Lumerical). The coupler dimensions were chosen to optimize mode matching and coupling efficiency with the LD output. These dimensions were selected within the feature size constraints of the fabrication process. As part of the design, the taper coupler was implemented in the partially etched SiN to reduce vertical (*Z*‐axis) optical confinement.

**FIGURE 3:**
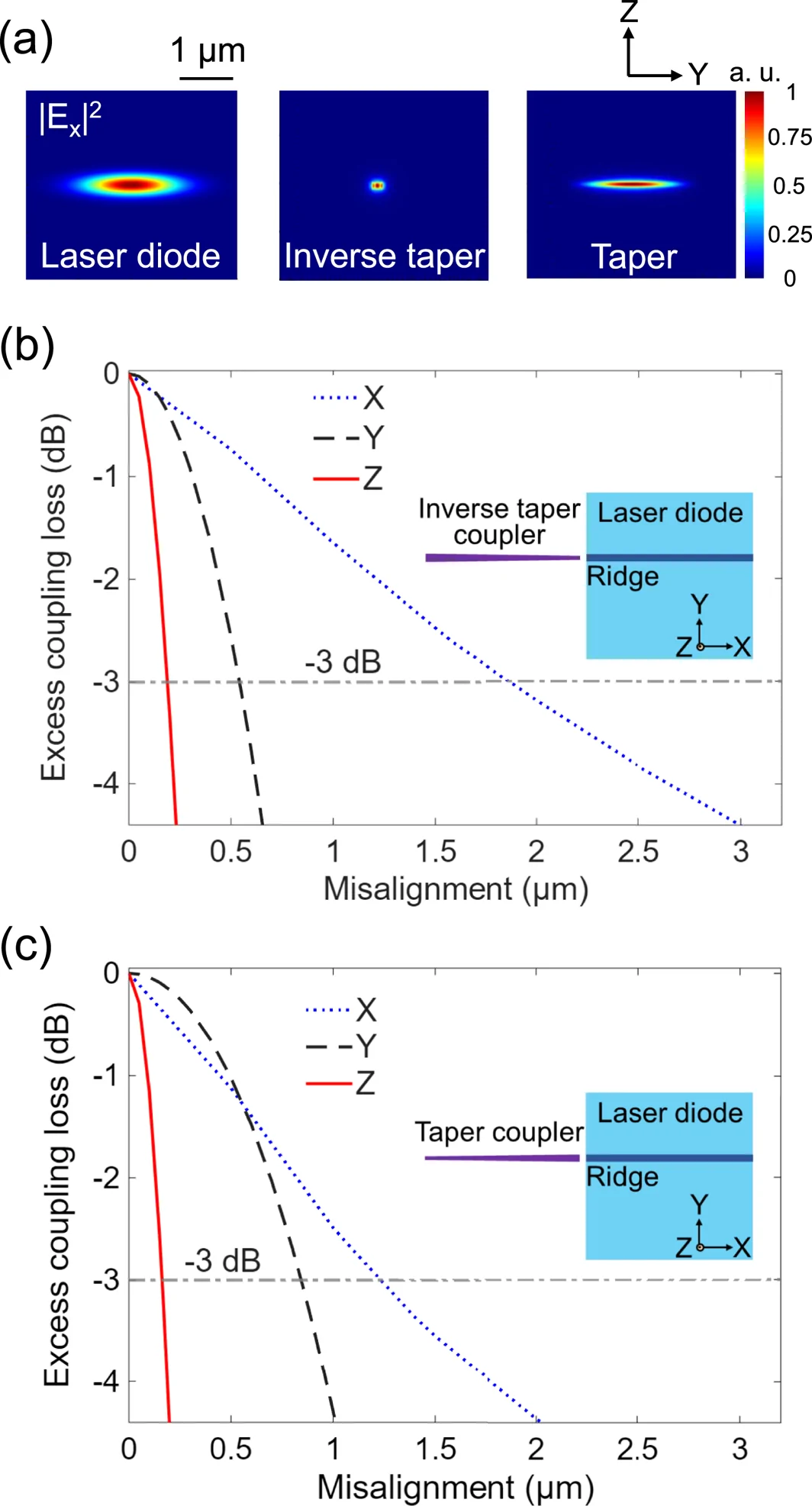
Simulated laser‐to‐waveguide coupling. (a) Optical mode profiles at the laser facet and the SiN coupler facet (both inverse taper and taper design); λ= 450 nm, transverse‐electric (TE) polarization. Simulated in‐plane misalignment tolerance of (b) inverse taper and (c) taper couplers. Insets: Illustrations of the LD‐to‐waveguide coupling (top‐down view).

Modal overlap calculations were performed to evaluate laser‐to‐waveguide coupling efficiency under ideal alignment conditions as well as with transverse (*Y*‐ and *Z*‐axis, Figures 1d and 3a) misalignment. Additional three‐dimensional finite‐difference time‐domain (FDTD) electromagnetic simulations (Ansys Lumerical) were performed to assess the impact of longitudinal (*X*‐axis) misalignment. With perfect alignment, the simulated coupling losses for the inverse taper and taper couplers are 2.9 dB and 0.6 dB, respectively. Excess coupling losses due to misalignment, relative to the perfectly aligned case, are shown in Figure 3b,c.

Table 1 summarizes the 3‐dB misalignment tolerances between the flip‐chip bonded LD and the SiN couplers on the Si photonic chip. Because the laser mode is more confined in the vertical direction (*Z*‐axis), optical coupling is more sensitive to vertical misalignment than the other two directions. Relative to the inverse taper, the taper coupler shows increased sensitivity to misalignment along the *X*‐ and *Z*‐axes, which is attributed to its reduced vertical mode size. By contrast, the taper coupler is more tolerant to *Y*‐axis misalignment because of its larger horizontal mode width. Overall, the taper coupler achieves lower coupling loss from simulation due to improved mode matching with the LD, while the inverse taper design offers greater tolerance to misalignment. For both coupler geometries, the misalignment tolerances are smaller than those typically reported for near‐infrared hybrid‐integrated lasers [30, 35], primarily due to the shorter wavelength (≈450 nm) and the correspondingly smaller optical mode size of the blue InGaN LD. The coupler design may be further optimized to improve misalignment tolerance, for example, through the use of multi‐tip geometries [36] or adiabatic couplers [41]. Additional simulation results for the rotational misalignment tolerances of both coupler designs are provided in Supporting Information S1: Figure S1.

**TABLE 1:** Summary of simulated 3‐dB misalignment tolerances of the LD‐to‐waveguide couplers along *X*, *Y*, and *Z* axes.

Coupler design	*X*	*Y*	*Z*
Inverse taper	1.87	0.55	0.20
Taper	1.23	0.85	0.16

*Note:* Unit: μm.

### 3.3 Bonding Process Characterization

To assess the repeatability of the flip‐chip bonding process, 40 laser bonding trials were performed (20 trials for each of the two LD‐to‐waveguide coupler designs). For each bonded LD, the post‐bonding in‐plane misalignment and the on‐chip optical power coupled into the SiN waveguide were measured. Due to the transparency of the InGaN LD substrate, the alignment marks on the LD and the Si photonic chip remained visible following bonding, Figure 4a. This property enabled in‐plane misalignment measurements via confocal laser scanning microscopy using a 3D surface profiler (Keyence VK‐X3000) to image the alignment marks. For each sample, the in‐plane misalignment was calculated as the average offset across the four alignment mark pairs.

**FIGURE 4:**
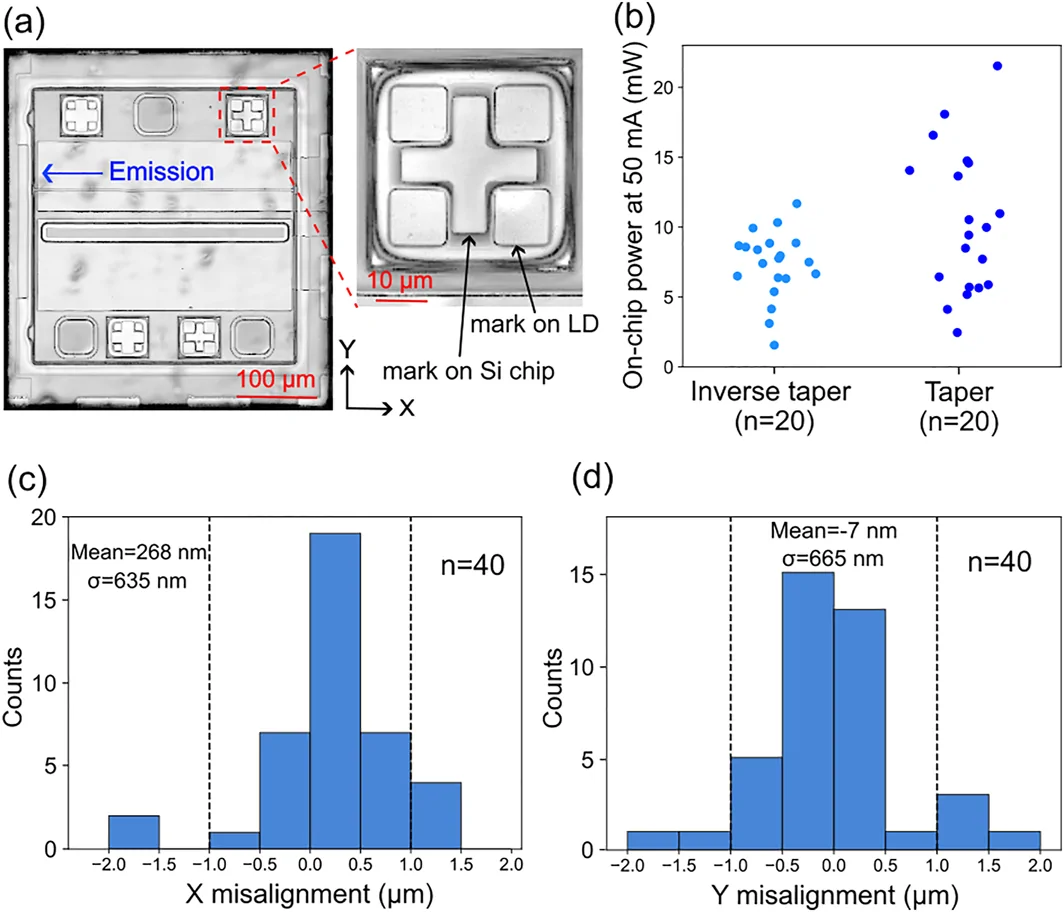
Characterization of the flip‐chip bonding process. (a) Confocal laser scanning micrograph of a representative flip‐chip bonded LD (left) with an enlarged view of one set of alignment marks (right). Cross: Si photonic chip mark; squares: LD mark; both visible through the LD. Micrograph quality was limited by imaging through the ≈100‐μm‐thick LD substrate. (b) On‐chip optical power distribution of flip‐chip bonded LDs (at 50‐mA drive current) with inverse taper and taper laser‐to‐waveguide couplers; *n* = 20 samples for each coupler design. Histograms of in‐plane misalignment after flip‐chip bonding along the (c) *X*‐ and (d) *Y*‐axis; *n* = 40; bin size = 0.5 μm; both coupler designs were included.

Histograms of the post‐bonding in‐plane misalignment along the *X*‐ and *Y*‐axes for all 40 samples are presented in Figure 4c,d, respectively. The measured misalignment exhibited standard deviations (σ) of 635 nm (*X*‐axis) and 665 nm (*Y*‐axis), with corresponding mean values of 268 nm and −7 nm, respectively. Misalignment within ±1 μm along both axes was observed for 85% of the bonded samples. The confocal laser scanning microscopy system exhibited a pixel‐resolution–limited measurement precision of ≈±140 nm, constrained by the reduced optical resolution from imaging through the ≈100‐μm‐thick InGaN LD substrate. Here, ≈±140 nm corresponds to the lateral sampling (pixel pitch) of the confocal images rather than the diffraction‐limited optical resolution. Misalignment is obtained from paired mark‐to‐reference distance measurements over micrometer‐scale spans, using edge identification and averaging over four alignment‐mark pairs to improve robustness, with the overall precision bounded by the pixel‐resolution limit. The post‐bonding laser‐to‐chip alignment precision was mainly limited by the placement accuracy of the die bonder. Furthermore, the use of a die bonder with a chip stage translation resolution of 1 μm introduced additional alignment constraints.

The angular tilt of the flip‐chip bonded LDs was characterized using white light interferometry with the 3D surface profiler (see Supporting Information S1: Figure S2). The vacant area adjacent to the bonding socket on the Si photonic chip served as the reference plane for the tilt measurements. The measured angular tilts of the flip‐chip bonded LD samples with both coupler designs are summarized in Table 2. According to the simulation results in Supporting Information S1: Figure S1, these angular deviations correspond to an estimated excess coupling loss of < 0.5 dB. Overall, post‐bonding rotational misalignment was minimal, as the alignment marks and mechanical stoppers effectively constrained angular tilt during flip‐chip bonding.

**TABLE 2:** Summary of measured rotational misalignment of the flip‐chip bonded samples.

Coupler design	Pitch	Roll	Yaw
Inverse taper	0.05°	0.05°	0.12°
Taper	0.07°	0.03°	0.20°

*Note:* The maximum angular tilt is reported. The definition of rotation axes is given in Figure 1d.

For characterization of the optical power, laser light coupled into each SiN waveguide was routed to an edge coupler at an output chip facet, coupled to a single‐mode optical fiber (Nufern S405‐XP) and measured with an optical power meter (Newport 2936‐R, 818‐SL/DB). Edge couplers at the output facet were nominally identical to the corresponding LD‐to‐waveguide couplers, using either inverse taper or taper designs. On‐chip optical power was calculated by subtracting the fiber‐to‐chip edge‐coupler loss (3.7 dB for inverse taper and 7.4 dB for taper couplers, measured separately using dedicated test structures) from the measured off‐chip, fiber‐coupled optical power. LDs bonded onto Si chips were characterized with the chips mounted on a copper heat sink atop a thermoelectric cooler (TEC) maintained at 21°C unless otherwise specified. Each LD was driven by a source measure unit (SMU, Keysight, B2912A), with current supplied through tungsten DC probes contacting the UBM pads on the Si photonic chip.

Figure 4b summarizes the measured on‐chip optical power from the 40 flip‐chip bonded LD samples. 85% of the LD samples with inverse taper couplers and 90% of those with taper couplers exhibited on‐chip optical power ≥ 5 mW. We attribute the variability in on‐chip power primarily to post‐bonding alignment, rather than to variations in LD emission power. This is consistent with measurements of 25 reference LDs from the same fabrication batch, mounted on TO‐56 headers instead of being flip‐chip bonded, which exhibited only a small emission power variation of ± 5.5% at a 50 mA drive current.

LD‐to‐waveguide coupling loss was determined by comparing the on‐chip optical power of flip‐chip‐bonded LDs (pulsed operation) with emission power of a reference LD (no flip‐chip process, pulsed operation) at identical drive currents. Pulsed operation reduced the effects of different heat sinking and also reduced the resulting variations in the operating temperature between the flip‐chip‐bonded and reference LDs, enabling accurate comparison. Twelve samples were selected for these additional measurements. The minimum laser‐to‐waveguide coupling loss was ≈4.1 dB for inverse taper couplers and ≈1.1 dB for taper couplers, corresponding to excess coupling losses (due to misalignment) of ≈1.2 dB and ≈0.5 dB, respectively (see Table 3). The on‐chip power distribution in Figure 4b is consistent with the simulation results in Section 3.2, confirming that relative to inverse taper couplers, taper couplers exhibit higher sensitivity to misalignment (indicated by a wider spread in measured power) but achieve higher coupling efficiency (reflected by higher maximum optical power levels). Because routing waveguide lengths differ between the LD test structures and the reference waveguides used to measure and de‐embed fiber‐to‐chip edge‐coupler loss, the extracted LD‐to‐waveguide coupling losses include residual SiN propagation loss and therefore represent an upper bound. The LD test structures contain up to 847.7 μm of additional waveguide length; using our prior measurement of 8.2 dB/cm loss for similar single‐mode SiN waveguides at 445 nm [42], the embedded propagation loss is estimated to be up to ≈0.7 dB. Subsequent platform development has reduced losses at 445 nm to approximately 3 dB/cm (not demonstrated here) [37].

**TABLE 3:** Summary of simulated and minimum measured coupling losses and measured on‐chip optical power for both laser‐to‐chip coupler designs.

Coupler design	Simulated loss	Measured loss (min.)	Measured on‐chip power (50 mA drive current)^a^	Measured on‐chip power (90 mA drive current)^a^
Inverse taper	2.9 dB	4.1 dB	7.5 ± 2.4 mW	11.6 ± 4.8 mW
Taper	0.6 dB	1.1 dB	10.5 ± 5.0 mW	19.0 ± 9.0 mW

^a^ Power represented by mean ±σ.

Table 3 summarizes the simulated and measured coupling losses together with the on‐chip optical power. The measured minimum coupling losses agree with the simulated results within 1.2 dB for the inverse taper and 0.5 dB for the taper, with the larger discrepancy for the inverse taper possibly arising from its increased sensitivity to small variations in tip width. The on‐chip optical power across samples showed significant variability, attributed to the LD‐to‐waveguide misalignment tolerance, which is comparable to the placement accuracy of the die bonder. The measured standard deviation of post‐bonding alignment along the *X*‐axis (635 nm) is 2.9 × smaller for the inverse taper and 1.9 × smaller for the taper than the simulated 3‐dB misalignment tolerance in Table 1. Along the *Y*‐axis, the standard deviation (665 nm) is 1.2 × larger for the inverse taper and 1.3 × smaller for the taper than the simulated tolerance. Although these values remain within the die bonder's specified placement accuracy, reducing variability in on‐chip optical power will require systems with higher precision. Section 4.3 provides estimates of coupling efficiency improvements achievable with sub‐300 nm precision, following Ref. [35].

The flip‐chip bonding process uses localized heating from the pickup tool to independently bond multiple LDs onto a single Si photonic chip (Figure 2d). When sockets are closely spaced, successive bonding steps may thermally affect previously bonded LDs, potentially altering their alignment. In addition, although the temperature at nearby sockets was kept below the AuSn solder melting point, thermal oxidation of the solder may still occur and reduce electrical conductivity [43]. To assess potential bonding‐order effects, we analyzed on‐chip optical power as a function of bonding sequence. Of the 40 integrated LDs, 26 were bonded onto Si chips hosting multiple LDs from the sample set; an extended analysis of their characterization results is presented in Supporting Information S1: Section S2.

For inverse taper couplers, the mean power of initially bonded LDs (exposed to thermal cycles from subsequent bonding steps) was 86% of that of last‐bonded LDs, whereas for taper couplers, the corresponding value was 64%. Comparison with Si chip samples on which only a single LD was bonded indicates that these mean values fall within the variability of the flip‐chip process. Thus, although the data suggest a possible influence of bonding order, any effect is difficult to distinguish from process variability. Improvements to the flip‐chip bonding process and LD‐to‐waveguide coupler design for reduced coupling variability may help clarify the significance of this effect. Additionally, increasing the bonding socket pitch could reduce thermal interactions between successive bonding steps. As an alternative to the thermocompression bonding used in this work, laser‐assisted flip‐chip bonding could provide highly localized heating with minimal thermal exposure, thereby reducing effects on adjacent bonded lasers during sequential bonding [44, 45].

## 4 Hybrid‐Integrated Laser Diodes

### 4.1 L‐I‐V Curves and Optical Spectra

The hybrid‐integrated laser diodes were characterized by measuring the optical power–current–voltage (L–I–V) curves and optical spectra under continuous‐wave (CW) operation. Figure 5a shows the L–I–V characteristics of a bonded LD aligned to a taper coupler on a Si photonic chip. This device achieved the highest measured peak on‐chip (SiN waveguide‐coupled) optical power in this work, 60.7 mW, before device failure, which was attributed to the high current density and device heating at a drive current of 185 mA. The maximum on‐chip wall‐plug efficiency (WPE), defined with respect to the on‐chip optical power, was ≈6.0% at a drive current of 150 mA and decreased to ≈5.3% at 185 mA for this sample. Supporting Information S1: Figure S4 presents additional L–I–V curves from two other LD samples (LD1 and LD2), where thermal rollover (without LD damage) was observed with drive currents exceeding 144 mA and 132 mA, respectively. LD1 and LD2 reached maximum on‐chip optical powers of 42.1 mW and 46.8 mW, respectively, with LD2 exhibiting the highest on‐chip WPE (≈7.8%) among the 40 bonded LD samples. The mean and standard deviation of on‐chip optical power across all samples at 50 mA and 90 mA drive currents are reported in Table 3.

**FIGURE 5:**
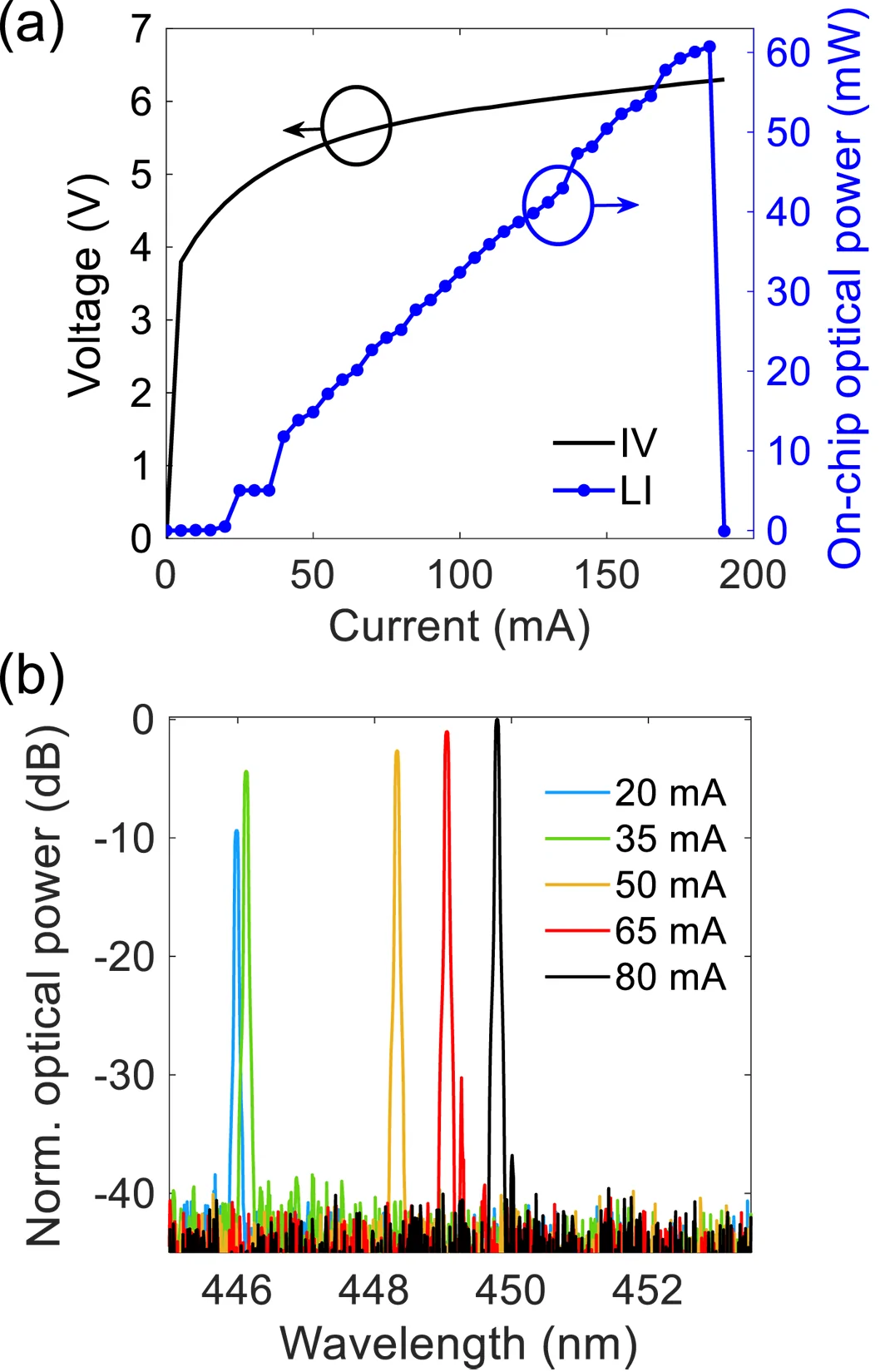
Characterization of a flip‐chip‐bonded laser diode showing the highest on‐chip optical power achieved in this work. (a) L‐I‐V curves measured in continuous‐wave (CW) mode. L: On‐chip optical power; I: drive current; V: voltage drop. Device failure occurred at a drive current of 185 mA. (b) Optical spectra at different laser drive currents (normalized to the peak power at 80 mA current).

Improvements in the on‐chip optical power may be realized through post‐assembly epoxy underfill, which decreases the large refractive index discontinuity in the gap between the LD and the SiN waveguide coupler, reducing reflections at the optical coupling interface. Additional improvement in optical power may be achieved through enhanced heat dissipation, for example, by employing thermally conductive epoxy encapsulation, larger laser bond pads and solder bumps [46], and/or a heat‐spreading layer with higher thermal conductivity [47]. Tests of LDs mounted in TO‐56 headers with full header contact yielded ≈2.4× average increases in thermal rollover current relative to the hybrid‐integrated LDs, indicating substantial potential for improved heat dissipation.

Additional tests were performed to evaluate the effects of epoxy underfill and encapsulation. Polydimethylsiloxane (PDMS, Sylgard 184) was applied at the laser‐to‐waveguide coupling interface, followed by a layer of thermally conductive epoxy (Cotronics, Duralco 128) on top of the LD to promote heat dissipation (see Supporting Information S1: Section S4). Across five samples, a moderate average on‐chip optical power increase of 12.6% was observed. Epoxy underfill and encapsulation were applied only in the tests shown in Supporting Information S1: Figure S5; unless otherwise specified, LD characterization was performed without these steps.

Figure 5b presents optical spectra of the flip‐chip‐bonded LD with the highest on‐chip optical power, measured at different drive currents with an optical spectrum analyzer (Yokogawa AQ6374). Emission peaks were centered near 450 nm, exhibiting a red shift with increasing drive current, consistent with laser heating at elevated drive currents. The measured spectral linewidth (full width at half maximum) remained within 0.05–0.10 nm, limited by the 0.05 nm resolution of the optical spectrum analyzer. At drive currents of 65 mA and 80 mA, multiple longitudinal modes were present, with side‐mode suppression ratios exceeding 29 dB.

Additional tests were conducted to examine the polarization properties of the LDs and to assess the impact of the flip‐chip bonding process on LD performance. Polarization was analyzed for both a flip‐chip‐bonded and a bare LD by sending the emitted light (from the Si photonic chip or bare LD) through a polarizer onto a free‐space detector (Newport 918D‐ST‐SL). Both LD samples were measured to be TE‐polarized, with polarization extinction ratios > 20 dB. Separately, the potential impact of the flip‐chip bonding process (heat and pressure) on the performance of the LD chips was evaluated by comparing the series resistance and emission power of the LDs before and after exposure to heat and pressure from the die bonder (see Supporting Information S1: Section S5). To enable direct measurement of LD emission power (rather than waveguide‐coupled power), the LDs were mounted contact‐side‐up on a blank Si test chip. Heat and pressure were then applied to the LD top surfaces via the pickup tool, with additional heating from the die bonder stage, following the same force and temperature profiles as in the full flip‐chip bonding process. Only minimal changes in LD performance were observed; averaged across three samples, the series resistance increased by ≈13.6% and the emission power increased by ≈13.3%. This slight increase in emission power may result from curing of the metal adhesive attaching the LD to the Si chip and the applied pressure, which together may have influenced thermal conduction.

### 4.2 Temperature Dependence

The emission power, voltage drop, and optical spectra of flip‐chip‐bonded LDs were measured over a temperature range from 14°C (above the dew point) to 80°C, with the heat sink temperature adjusted by a TEC controller (Arroyo Instruments, 5240 Series). Figure 6 shows the L–I–V curves and optical spectra of a representative LD sample (different from that in Figure 5). The L–I curves in Figure 6a indicate an increase in threshold current (from 16 mA to 22 mA) and a decrease in on‐chip optical power (≈15.5% drop at an 80 mA drive current) as the heat sink temperature was increased. The relatively small optical power reduction at elevated temperatures with high drive currents is attributed to efficient heat sinking through the direct thermal path from the LD metal contacts to the Si substrate (via the AuSn solder and UBM). The characteristic temperature of this LD sample, calculated from the measured L–I data, was estimated to be 187 K, consistent with previously reported values for blue InGaN laser diodes with emission wavelengths between 440 nm and 450 nm [48, 49]. Additionally, the inset in Figure 6a shows that the voltage across the LD decreased with increasing temperature, with a ≈13.6% drop at an 80 mA drive current, aligning with the temperature‐dependent voltage behavior of blue InGaN LDs reported in Refs. [50, 51].

**FIGURE 6:**
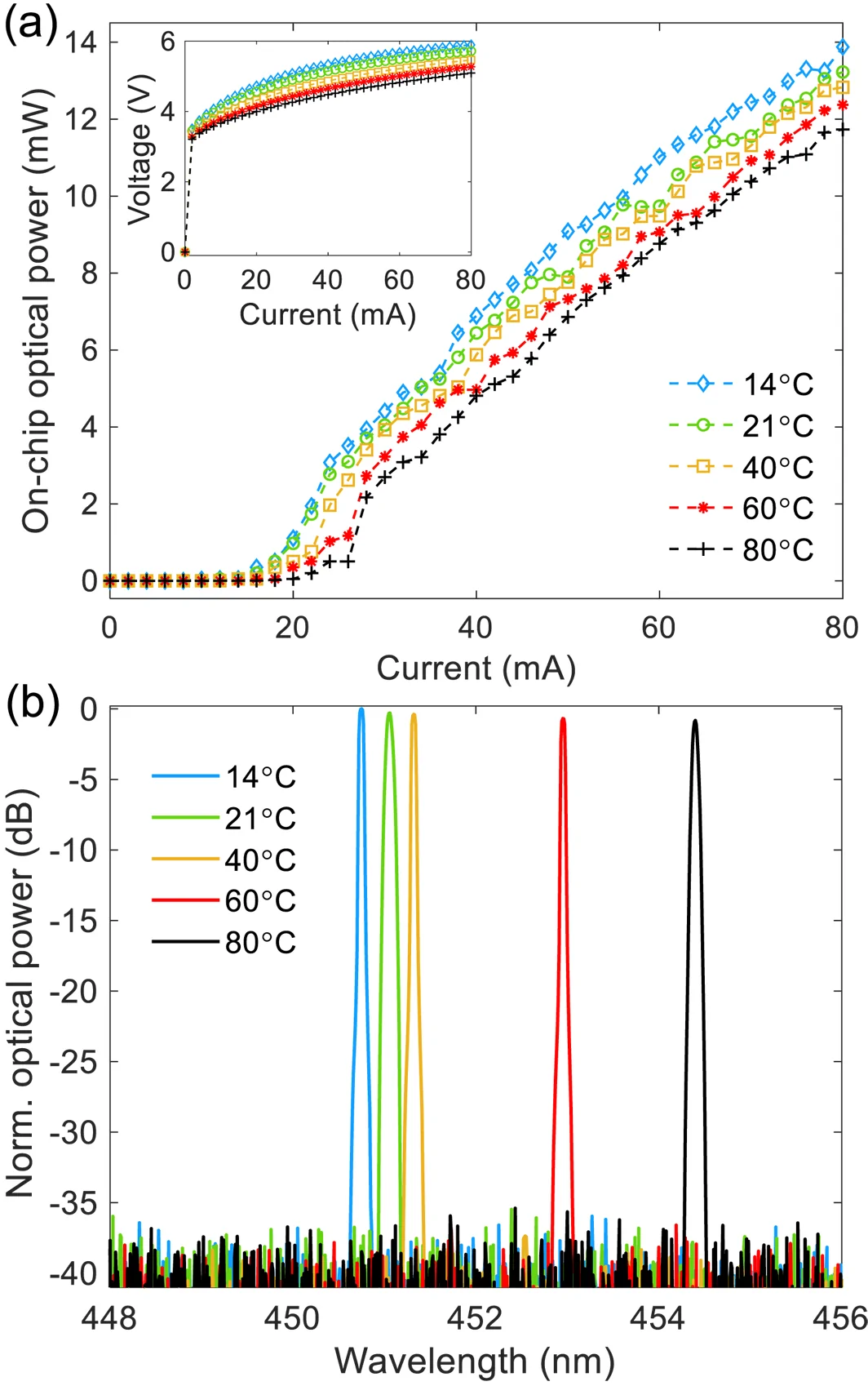
Temperature‐dependent performance of a representative flip‐chip bonded laser diode. (a) L‐I curves at various temperatures. (Inset) Corresponding I‐V curves. The L‐I‐V curves were averaged over 3 measurements. (b) Optical spectrum at temperatures corresponding to (a); 80‐mA drive current, normalized to the peak power at 14°C.

Lastly, Figure 6b presents the temperature dependence of the flip‐chip‐bonded LD emission spectrum. A red shift in the peak wavelength was observed as the heat sink temperature increased. The spectral linewidth remained between 0.05 nm and 0.10 nm over the tested temperature range. In agreement with Figure 6a, a small decrease in peak optical power was observed at higher temperatures.

### 4.3 Performance Summary and Comparison

The flip‐chip‐bonded, single‐spatial‐mode, 450‐nm InGaN laser diodes achieved high waveguide‐coupled on‐chip optical powers of up to 60.7 mW and on‐chip wall‐plug efficiencies up to 7.8%. The series resistance and emission power of the LDs showed only minor changes after flip‐chip assembly. Together, these results demonstrate the high peak performance achievable with this technique while indicating minimal damage to the LDs during hybrid integration. The primary limitation of this demonstration is the variability in post‐bonding alignment and thus on‐chip optical power, which constrains the repeatability and assembly yield of the current passive‐alignment flip‐chip bonding approach. Improvements in the assembly yield are expected using die bonders with higher placement accuracy and laser‐to‐waveguide coupler designs with larger misalignment tolerance. Based on the simulated alignment sensitivities in Figure 3, improving placement precisions from the standard deviations reported in Figure 4 (635 nm for *X*‐axis and 665 nm for *Y*‐axis) to 300 nm (the placement accuracy of the die bonder used in Ref. [35]) would reduce the excess coupling loss by 0.16 dB (*X*‐axis) and 2.1 dB (*Y*‐axis) for inverse taper couplers and by 0.17 dB (*X*‐axis) and 1.3 dB (*Y*‐axis) for taper couplers. Additionally, multi‐tip edge couplers have been reported for the visible spectrum, with simulated 1‐dB in‐plane misalignment tolerances of 2.6 μm (lateral, *Y*‐axis) and 3.5 μm (longitudinal, *X*‐axis) [36], offering substantial improvements over the tolerances listed in Table 1.

Table 4 compares this work with other reported hybrid laser integration demonstrations using passive‐alignment flip‐chip bonding at both visible and near‐infrared wavelengths. Despite higher laser‐to‐waveguide alignment sensitivity at shorter wavelengths (where optical mode dimensions decrease) in addition to the limited placement accuracy of our die bonder, this work presents on‐chip optical powers of up to 60.7 mW and a minimum coupling loss of 1.1 dB with flip‐chip‐bonded blue LDs. These results are comparable to the best reported performance achieved with passive‐alignment flip‐chip bonding technology at near‐infrared wavelengths [34]. In comparison to similar demonstrations in the visible spectrum [7, 36], this work achieves ≳ 6× higher on‐chip optical power.

**TABLE 4:** Comparison of hybrid laser/amplifier integration demonstrations using passive‐alignment flip‐chip bonding.

Reference	LD/SOA type	Photonic waveguide	Wavelength (nm)	Placement accuracy	Max. CW on‐chip optical power	Min. coupling loss
[52]	LD	Si	Near infrared	Sub‐micron	≈3 mW	7 dB
[34]	InP DFB LD	SiN	≈1550	Sub‐micron	≈40 mW	3 dB^a^
[35]	InP DFB LD	SiN	≈1550	Sub‐300 nm	40 mW (with epoxy underfill)	1.1 dB
[36]	GaAs SOA	SiN	≈640	Sub‐2 μm	Lasing operation not achieved	7.1 dB
[7]	RGB LDs	SiN	RGB	Sub‐500 nm	≈10 mW^b^	N.A.
This work	InGaN LD	SiN	≈450	Sub‐micron	60.7 mW	1.1 dB

Abbreviations: DFB, distributed feedback; GaAs, gallium arsenide; InP, indium phosphide; RGB, red, green, and blue; SOA, semiconductor optical amplifier.

^a^ Results for one sample reported.

^b^ Free‐space emission from the integrated photonic chip, combining 3 LDs (R, G, B).

## 5 Photonic Integrated Circuit Demonstration

In this section, we present a proof‐of‐concept active photonic integrated circuit combining our Si photonic platform's thermo‐optic switching and photodetection capabilities with a hybrid‐integrated InGaN LD. An LD was flip‐chip bonded into a socket on the PIC following the method in Section 3.1. Emitted light was routed through a SiN power tap that diverted a small fraction to a waveguide‐coupled photodetector for power monitoring, followed by a thermo‐optic switch (Figure 7a). The power tap was implemented using an evanescent directional coupler with a designed power coupling of ≈1%. A 200‐nm‐wide SiN waveguide was routed underneath a 200‐μm‐long Si PIN junction to form the photodetector [39]. The optical switch used a Mach–Zehnder interferometer configuration with a 324‐μm‐long TO phase shifter in each arm. The TO phase shifter featured a TiN heater along with a 3‐pass folded waveguide and thermal isolation trenches to enhance phase modulation efficiency (Figure 1a); no undercut etching was performed. The switch supported optical switching between two output ports (Outputs 1 and 2), as well as variable optical attenuation at one selected output port. The output ports were routed to edge couplers at a chip facet for coupling to a single‐mode optical fiber. Tungsten needle probes contacted the UBM and aluminum pads on the Si photonic chip to apply electrical drive signals and measure photocurrent.

**FIGURE 7:**
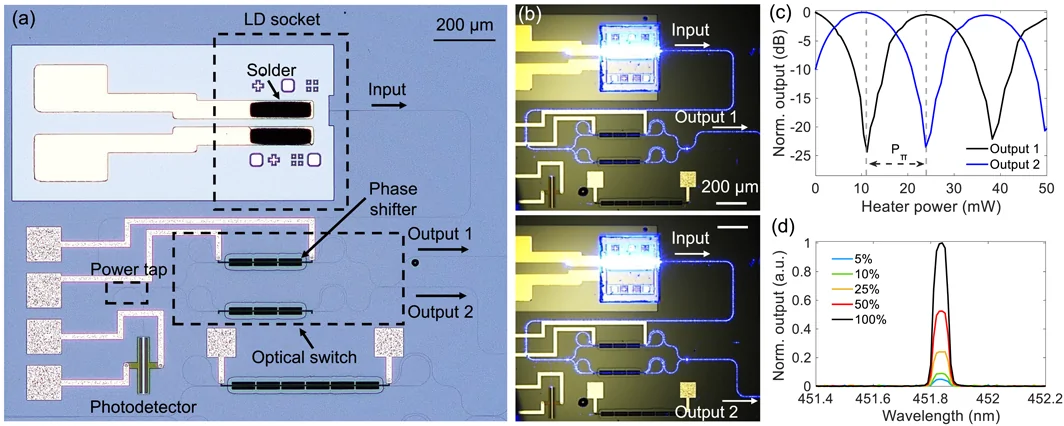
Photonic integrated circuit (PIC) with a flip‐chip bonded LD, an integrated photodetector, and a thermo‐optic (TO) switch. (a) Micrograph of the PIC prior to laser bonding. (b) Micrographs of the PIC (after laser bonding) showing operation of the bonded laser and switching between the output waveguides (Outputs 1 and 2). (c) TO switching operation: normalized output optical power from the PIC versus heater power dissipation of the TO phase shifter. (d) Variable optical attenuation using the switch: normalized optical spectra of PIC Output 1 at various optical power levels. The laser drive current was 35 mA.

### 5.1 Optical Switching and Variable Attenuation

Figure 7b demonstrates the optical switching operation of the PIC, achieved by driving the LD and the TO phase shifter. The normalized PIC optical output power as a function of heater power dissipation is plotted in Figure 7c. The extinction ratio (ER) of the optical switch and heater power for a π‐radian phase shift ($P_{\pi }$) in the phase shifter were calculated from the first half‐period of Figure 7c. Outputs 1 and 2 exhibited ERs of 24.4 dB and 23.5 dB, with corresponding $P_{\pi }$ values of 12.7 mW and 13.4 mW, respectively. Although the switching speed was not characterized in this work, a similar TO switch has been reported to exhibit a switching speed of 11.8 kHz [53].

The optical switch also functions as a variable optical attenuator (VOA) when a single output port is used. This functionality is valuable in applications requiring precise, high‐dynamic‐range optical power control without associated wavelength shifts, such as laser‐based displays. Such control is difficult to achieve through LD current tuning because of the dependence of wavelength on current and the nonlinearity of the L–I characteristics near threshold, Figure 5. To demonstrate VOA operation with the PIC, the LD was driven at a constant current of 35 mA, while the heater power applied to the phase shifter was varied to control the optical power at Output 1. Figure 7d shows the normalized optical spectra from Output 1 at different power levels relative to the peak optical power. The peak emission wavelength (≈451.8 nm) and spectral linewidth (0.05–0.10 nm) remained stable across all measured optical power levels (spanning 13 dB of attenuation), owing to the constant LD drive current.

### 5.2 On‐Chip Power Monitoring

The active functionality of the PIC was further tested through on‐chip power monitoring of the flip‐chip‐bonded LD emission using the integrated photodetector. The photocurrent from the on‐chip photodetector was characterized under both DC and AC LD driving conditions. DC power‐monitoring characteristics are shown in Figure 8a, comparing on‐chip photocurrent with off‐chip power measurements as the LD drive current was varied. The traces are normalized to facilitate comparison of their shapes. The photocurrent and optical power curves are consistent, both exhibiting high linearity above the lasing threshold.

**FIGURE 8:**
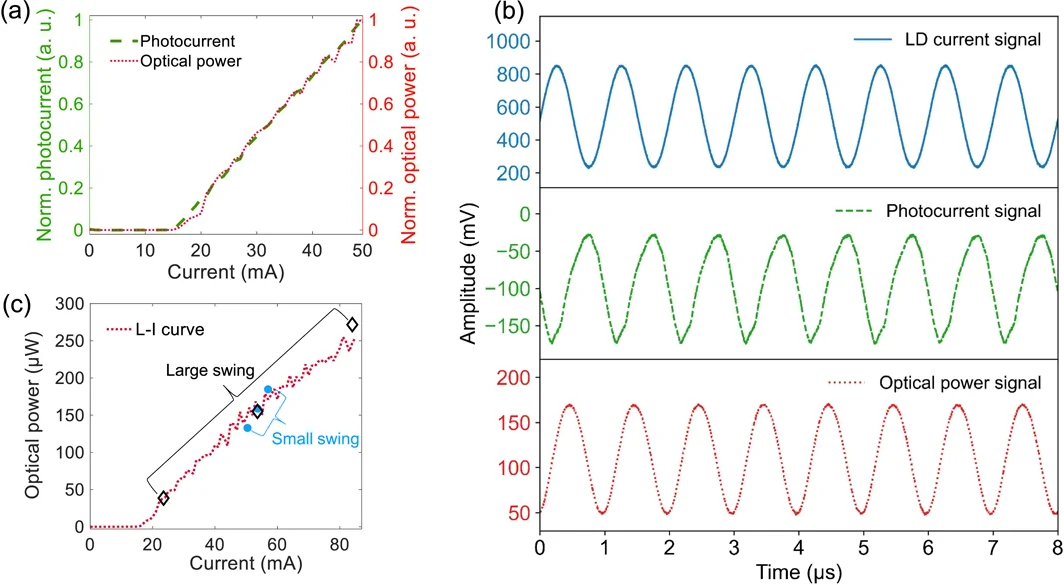
PIC power monitoring demonstration using the integrated photodetector in Figure 7a. (a) Normalized DC photocurrent and output optical power from the PIC versus LD drive current; data were averaged over 7 measurements. (b) Comparison of signals acquired by the oscilloscope during sinusoidal LD current modulation at 1 MHz. (c) Comparison of off‐chip optical power with DC drive current (L‐I curve) and dynamic optical power during LD modulation (maximum, average, and minimum of the sinusoidal modulation). The L‐I curve was averaged over 4 measurements. Small‐ and large‐swing LD modulation cases are shown, distinguished by colored brackets.

For AC modulation, the on‐chip photocurrent was compared with the LD drive current waveform and with a reference off‐chip optical power measurement recorded using an external detector. In these measurements, the LD was DC‐biased via a source measure unit and modulated with a 1 MHz sine wave AC signal from an arbitrary function generator (AFG, Tektronix AFG31000). The DC bias and AC modulation signals were combined using a bias tee (Pasternack, PE1BT1002) to drive the LD, with a 10‐Ω sensing resistor connected in series to measure the current during modulation. The integrated photodetector was reverse‐biased at 2 V using a second channel of the source measure unit, and its photocurrent was amplified by a transimpedance amplifier (TIA, Koheron TIA100‐2k‐SMA). The modulated optical output of the PIC was collected through a single‐mode fiber and detected by an off‐chip Si switchable gain detector (Thorlabs, PDA36A2). The LD current (measured via the sensing resistor in series), photocurrent from the integrated photodetector, and off‐chip optical power were recorded simultaneously with an oscilloscope (Keysight, DSOX4054A). A schematic of the measurement setup is shown in Supporting Information S1: Figure S7.

During modulation, the LD was biased at a DC current of 53 mA to ensure stable emission above the lasing threshold while avoiding thermal rollover within the modulation swing. Both small and large current swings were tested, with AFG voltage amplitudes of 0.5 $V_{\mathrm{pp}}$ and 4.5 $V_{\mathrm{pp}}$, respectively. The measured LD current, on‐chip photocurrent, and off‐chip optical power signals during LD modulation for the large current swing case are shown in Figure 8b. The photocurrent signal was synchronized with the LD current signal, exhibiting a phase inversion attributed to the inverting operation of the TIA. The off‐chip optical power is shown in the bottom panel of Figure 8b, with a slight phase shift relative to the LD current signal, likely due to the internal phase delay of the switchable gain detector. All three signals maintained a consistent frequency of 1 MHz with sinusoidal waveforms.

Calculations and calibration measurements were used to determine the amplitudes of the LD current and off‐chip optical power during modulation from the oscilloscope voltage traces. Using the known sensing resistor value, the LD current was found to vary from ≈23.5 mA to ≈84.0 mA for the large current swing and from ≈50.4 mA to ≈57.0 mA for the small current swing. Next, the procedure described in Supporting Information S1: Section S7 was applied to extract the magnitude of the optical power modulation measured by the off‐chip detector (referred to as the dynamic optical power). Figure 8c compares the DC off‐chip optical power versus current (static L–I curve averaged over 4 measurements) with the maximum, average, and minimum off‐chip optical powers during AC modulation. Both the small and large current swing cases are shown in Figure 8c. The derived dynamic power values closely follow the static L–I curve and indicate optical power modulations (ratio of maximum to minimum) of ≈7.1× and ≈1.4× for the large and small current swings, respectively. Small differences between static and dynamic optical powers arise from the inherent LD temperature variations in these operating conditions.

### 5.3 Integration Outlook

The demonstrated integration of visible‐light LDs with on‐chip thermo‐optic tuning and photodetection provides a practical and fundamental set of functionalities for visible‐spectrum PICs, particularly for enabling wavelength‐tunable lasers, which have diverse applications such as quantum information processing [54], spectroscopy [55], and biosensing [56]. Recent work on visible‐light LDs and gain chips actively aligned to SiN PICs have demonstrated wavelength tunability via thermo‐optically controlled feedback [31, 32]. The integration of photodetectors, as demonstrated here, opens new opportunities for fully‐integrated wavelength‐tunable lasers. In principle, these on‐chip devices would allow stabilization and tracking of both emission power (through power taps) and wavelength (through integrated wavemeters). Additionally, the building blocks integrated in this work are directly applicable to emerging PIC‐based microdisplays for augmented and virtual reality [6, 7]. In such systems, on‐chip switches and VOAs could enable compact, dynamic routing to different emitters and brightness control, while integrated photodetectors could facilitate brightness monitoring, which is particularly important for ensuring eye safety. In the above applications, passive‐alignment flip‐chip LD bonding, combined with foundry‐based Si photonics fabrication, offers a direct path to scaling PIC complexity, wafer fabrication volumes, and assembly throughput.

As shown in our recent work [26], similar integrated photodetectors on this platform achieve up to 18 GHz bandwidth and support avalanche gain. The 1 MHz AC modulation tested in Section 5.2 was limited by the bandwidths of the TIA (2 MHz) and the switchable‐gain detector used for off‐chip power measurements (1.6 MHz at 10 dB gain), not by the device itself. Future implementations of PICs incorporating arrays of directly‐modulated, hybrid‐integrated, visible‐spectrum LDs, together with high‐bandwidth, sensitive photodetectors, could form the basis of new microsystems for short‐reach interconnects [57] and underwater communications [15].

Though not demonstrated here, our visible‐light Si photonic platform also supports microelectromechanical systems (MEMS) devices for beam scanning. These can be realized either by undercut etching the Si substrate to release movable ${\text{SiO}}_{2}$‐cladding cantilevers or by wafer thinning to define larger structures with a robust Si backbone. Integrating waveguide emitters into these devices enables compact beam scanners; we have recently demonstrated both electrothermally actuated ${\text{SiO}}_{2}$ scanners [58] as well as Si MEMS scanners driven by co‐packaged piezoelectric actuators [19]. Integration of these devices with on‐chip lasers would enable compact, fully integrated beam‐scanning systems, opening new possibilities for applications such as next‐generation microdisplays and advanced scanning microscopy systems [59].

## 6 Conclusion

In summary, we have demonstrated the hybrid integration of blue, 450‐nm–wavelength, InGaN laser diodes onto a foundry‐fabricated visible‐light Si photonics platform using passive‐alignment flip‐chip bonding. Leveraging the co‐design of the laser diodes and Si photonic chips, which included precisely patterned alignment marks and mechanical stoppers, the flip‐chip bonding process achieved sub‐micron in‐plane laser‐to‐chip alignment accuracy. Across the 40 flip‐chip–bonded laser samples, the measured optical power coupled to the Si photonic chip reached 60.7 mW. A highest on‐chip wall‐plug efficiency of 7.8% and a minimum coupling loss of 1.1 dB were achieved. Thermal characterization showed stable operation of the flip‐chip–bonded lasers at elevated temperatures up to 80°C, maintaining high optical output power. A proof‐of‐concept active photonic integrated circuit was demonstrated, incorporating a flip‐chip bonded laser diode, an integrated waveguide‐coupled Si photodetector as an on‐chip power monitor, and a thermo‐optic switch for dynamic optical routing and variable optical attenuation.

Overall, this work establishes a new benchmark for scalable hybrid integration of visible‐wavelength laser diodes on Si photonic platforms. Although challenges remain in reducing variability in laser‐to‐chip alignment, advances in high‐precision die bonding and laser‐to‐waveguide coupler designs present clear pathways for further improvement. The localized heating used in the flip‐chip bonding method is compatible with integrating multiple laser diodes on a single PIC, and future efforts will target incorporating lasers at multiple wavelengths. We anticipate that the combined functionalities of our platform, including hybrid‐integrated lasers, thermo‐optic devices, photodetectors, and MEMS devices, will enable fully‐integrated wavelength‐tunable laser arrays, as well as complex PICs for applications in augmented and virtual reality, quantum information processing, biophotonics, and data communications.

## Author Contributions

**Xin Mu:** writing – original draft, visualization, writing – review and editing, validation, investigation, formal analysis, methodology, software. **Frank Weiss:** investigation, methodology, validation. **Hongyao Chua:** methodology, investigation. **Robert Lawrowski:** methodology, investigation, visualization. **Jared C. Mikkelsen:** investigation. **John N. Straguzzi:** investigation. **Hannes Wahn:** investigation, writing – review and editing. **Piyush Kumar:** investigation, writing – review and editing. **Guo‐Qiang Lo:** supervision, resources, project administration. **Joyce K. S. Poon:** supervision, funding acquisition, resources, writing review and editing, conceptualization. **Mariel Jama:** methodology, investigation, conceptualization, supervision, funding acquisition, resources, visualization, project administration, writing – review and editing. **Wesley D. Sacher:** supervision, conceptualization, writing – original draft, funding acquisition, methodology, investigation, project administration, writing – review and editing, resources.

## Funding

This work was supported by the Max Planck Society. The authors Robert Lawrowski and Mariel Jama gratefully acknowledge partial financial support by the German Federal Ministry of Education and Research (BMBF) in the funding program Trusted Electronics (ZEUS) within the joint project VE‐Silhouette under grant ME1ZEUS006.

## Conflicts of Interest

The authors declare no conflicts of interest.

## Supporting information


Supporting Information S1

## Data Availability

The datasets generated during and/or analyzed during the current study are available from the corresponding authors on reasonable request.
