# Supplementary material for: Hybrid Integration of InGaN Lasers in a Foundry‐Fabricated Visible‐Light Photonics Platform
Source: Nanophotonics. 2026 Apr 10;15(7):e70037. doi: 10.1002/nap2.70037 (PMC13642095; doi:10.1002/nap2.70037)
Supplement: Supplementary file 1 [file NAP2-15-e70037-s001.pdf]

Research Article

Xin Mu*, Frank Weiss, Hongyao Chua, Robert Lawrowski, Jared C. Mikkelsen, John N. Straguzzi, Hannes Wahn, Piyush Kumar, Guo-Qiang Lo, Joyce K. S. Poon, Mariel Jama, and Wesley D. Sacher*

Hybrid integration of InGaN lasers in a foundry-fabricated visible-light photonics platform

Supplementary materials

S1 Rotational misalignment of flip-chip bonded laser diodes

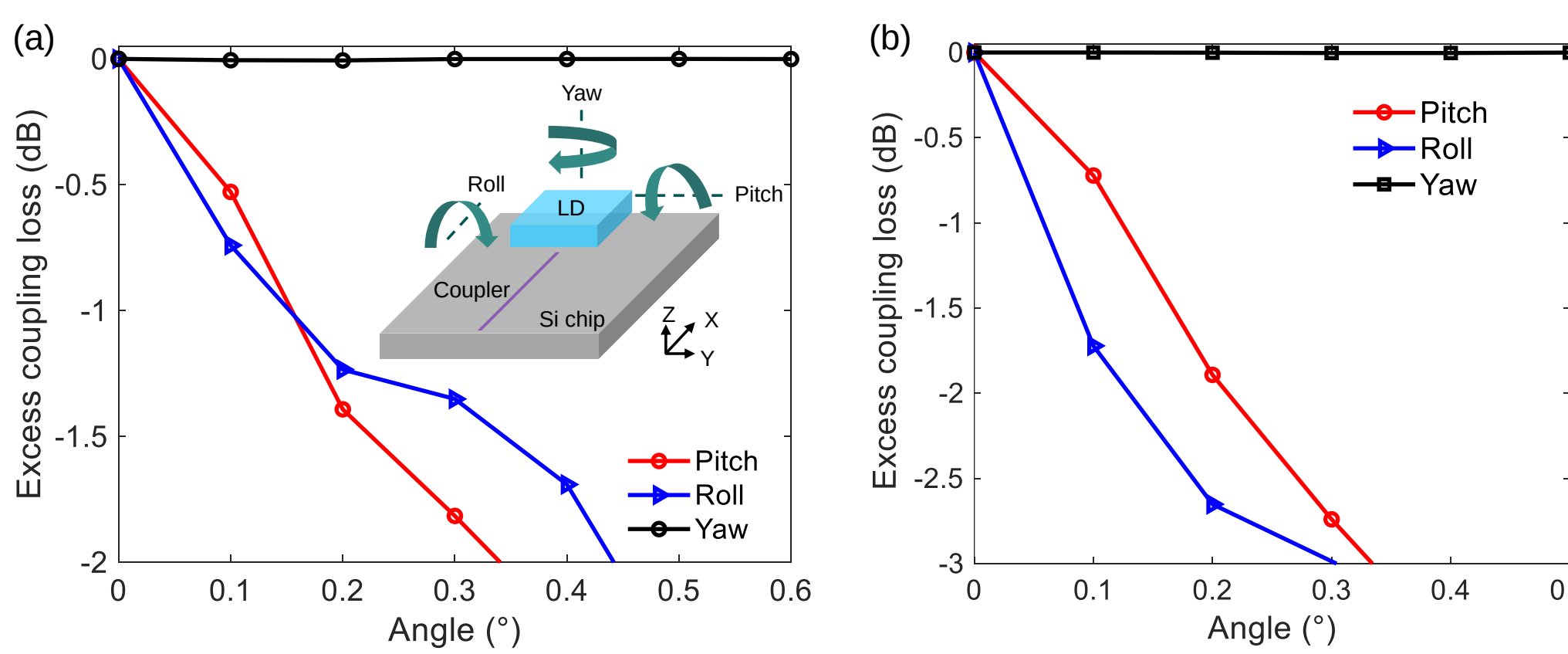

Figure S1: Simulation of rotational misalignment between the laser diodes (LDs) and silicon nitride (SiN) waveguide couplers for (a) inverse taper and (b) taper coupler designs. Inset: illustration of the rotation axes.

To investigate the influence of rotational misalignment between the laser diode (LD) and the silicon nitride (SiN) waveguide coupler, we simulated the excess coupling loss caused by angular tilts along the pitch, roll, and yaw axes, as shown in the inset of Figure S1(a). The coupling loss was calculated with three-dimensional finite-difference time-domain (FDTD) electromagnetic simulations (Ansys Lumerical). The simulations indicate

***Corresponding authors: Xin Mu, Wesley D. Sacher,**
Xin Mu, Max Planck Institute of Microstructure Physics, Weinberg 2, 06120 Halle, Germany; and University of Toronto, 10 King's College Road, Toronto, Ontario, M5S 3G4, Canada, E-mail: xinmu@mpi-halle.mpg.de. https://orcid.org/0000-0001-6188-7969; and **Wesley D. Sacher**, Max Planck Institute of Microstructure Physics, Weinberg 2, 06120 Halle, Germany, E-mail: wesley.sacher@mpi-halle.mpg.de.
Frank Weiss, Jared C. Mikkelsen, John N. Straguzzi, Hannes Wahn, Piyush Kumar, Max Planck Institute of Microstructure Physics, Weinberg 2, 06120 Halle, Germany
Joyce K. S. Poon, University of Toronto, 10 King's College Road, Toronto, Ontario, M5S 3G4, Canada
Hongyao Chua, Guo-Qiang Lo, Advanced Micro Foundry Pte. Ltd., 11 Science Park Road, Singapore Science Park II, 117685, Singapore
Robert Lawrowski, Mariel Jama, ams OSRAM International GmbH, Leibnizstrasse 4, 93055 Regensburg, Germany

that taper couplers in Figure S1(b) are more sensitive to rotational misalignment compared to inverse taper couplers in Figure S1(a). As detailed in Section 3.3, among the 40 flip-chip bonded LD samples, the measured angular tilts were minimal, corresponding to simulated excess coupling losses of <0.5 dB along all three rotation axes for both coupler designs.

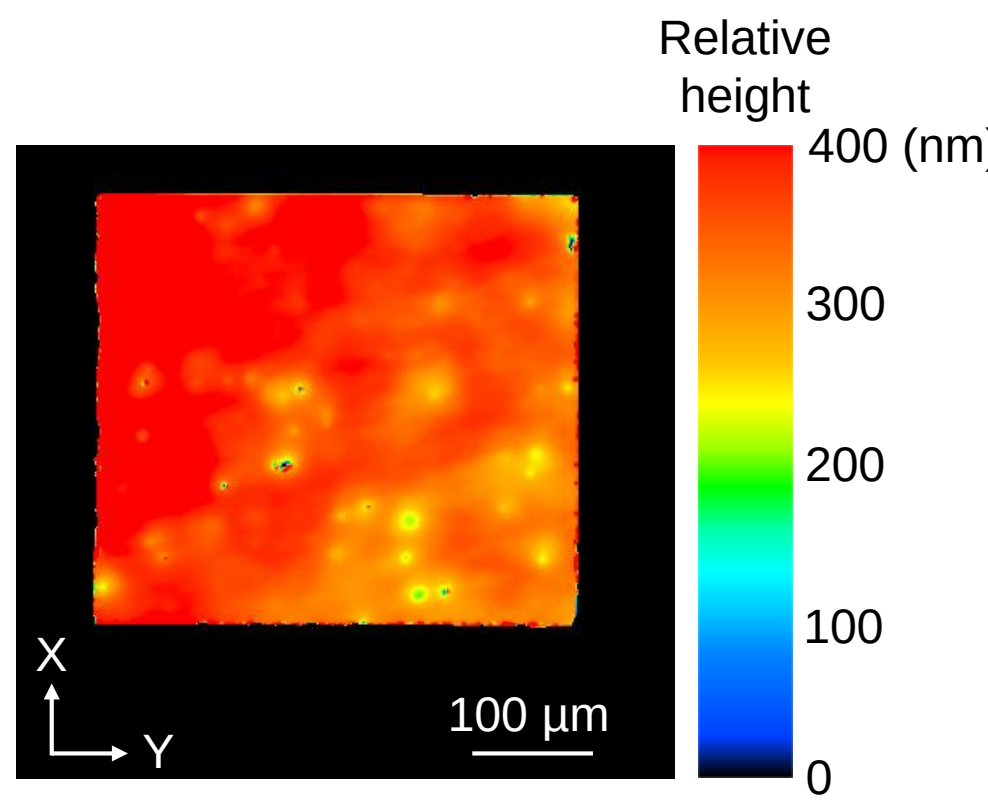

Figure S2: Height profile measurement of a flip-chip bonded laser diode using white light interferometry. The height offset between the reference plane on the silicon (Si) photonic chip (shown in black) and the top surface of the laser diode (colored) was subtracted to show the relative height difference. The displayed height range was adjusted to improve visibility of the measured relative height difference.

We also evaluated the rotational misalignment [pitch and roll axes; yaw-axis tilt calculated based on the 4 alignment marks pairs as shown in Figure 4(a)] by measuring the height of flip-chip bonded laser diodes. Figure S2 shows the top surface profile of a representative flip-chip bonded laser diode. The height was measured with white light interferometry using a 3D surface profiler (Keyence, VK-X3000). The vacant area near the measured LD bonding socket on the silicon (Si) chip served as the reference plane. The upper and lower limit of the displayed heights were adjusted for better visibility of the height variation on the LD, which was small compared to the ≈100 µm LD thickness. In addition, the height offset between the reference plane on the Si chip and the top surface of laser diode was subtracted during the measurements, showing the relative height difference. The maximum height difference along the X and Y axes of the LD shown in Figure S2 are ≈130 nm and ≈180 nm, respectively, corresponding to angular tilts of <0.02° (pitch) and <0.03° (roll). The yaw-axis tilt was measured as 0.04° for this sample.

S2 Bonding of multiple LDs on a single Si photonic chip

Localized heating from the pickup tool in the flip-chip bonding process enables independent bonding of multiple LDs onto a single Si photonic chip. However, when bonding sockets are in close proximity, thermal exposure from successive bonding steps may influence the alignment of previously bonded LDs. To assess this potential impact, additional data analysis is presented below.

The Si photonic chips used to test flip-chip bonding of LDs (Sections 3 and 4) included multiple bonding sockets, Figure 2(d). Each chip contained two rows of sockets: one designed with inverse tapers and the other with tapered laser-to-waveguide couplers. The intra-row pitch was 640 µm, while the inter-row pitch was larger at 2.8 mm. Among the 40 bonded LD samples shown in Figure 4(b), 26 were associated with Si photonic chips onto which 2 − 3 LDs had been bonded in the same row. Of these, 8 samples used bonding sockets with inverse-taper couplers and 18 used taper couplers. In the remaining cases, only a single LD was bonded per Si chip. Characterization of the flip-chip bonded LDs occurred after all LDs had been bonded to the corresponding Si chip.

Evaluation of the measured on-chip optical powers with respect to the bonding sequence provides insight into the dependence of integrated LD performance on subsequent bonding steps. Figure S3 shows the on-chip

optical powers of these samples, which represent a subset of the data presented in Figure 4(b) (measured at an LD drive current of 50 mA). The data are categorized by Si photonic chips with LDs bonded to sockets incorporating inverse taper couplers (Chips 1–2) and tapered laser-to-waveguide couplers (Chips 4–10). On Chip 3, LDs were bonded to both rows of sockets; the corresponding data are labeled as Chip 3a and Chip 3b.

Figure S3 compares the on-chip optical power of LDs as a function of bonding order, where the first-bonded LDs in each sequence were subsequently exposed to thermal cycles from later bonding steps. For inverse taper couplers, the mean power of initially bonded LDs was 86% of that of last-bonded LDs. For taper couplers, the first-bonded LDs exhibited a mean power only 64% of that of last-bonded LDs. Additionally, for the complementary set of samples (not shown in Figure S3) in which only a single LD was bonded per Si photonic chip, the mean on-chip optical powers (± standard deviation) were 8.1 ± 2.6 mW for inverse taper couplers and 9.0 ± 4.7 mW for taper couplers. With respect to Figure S3, these values align with the mean of last-bonded LDs for inverse taper couplers and, conversely, with the mean of first-bonded LDs for taper couplers, highlighting the variability inherent to the bonding process.

While the data in Figure S3 point to a potential influence of bonding sequence on LD performance, the observed differences are comparable to the variability seen in chips with only a single bonded LD. Refinements to the flip-chip process and coupler design, aimed at reducing coupling variability, are necessary to determine whether such effects are significant. Increasing the pitch between bonding sockets is expected to reduce thermal crosstalk between successive bonding steps. Alternatively, laser-assisted bonding can confine heating to a small volume, reducing overall thermal exposure and minimizing unintended effects on adjacent, previously bonded lasers.

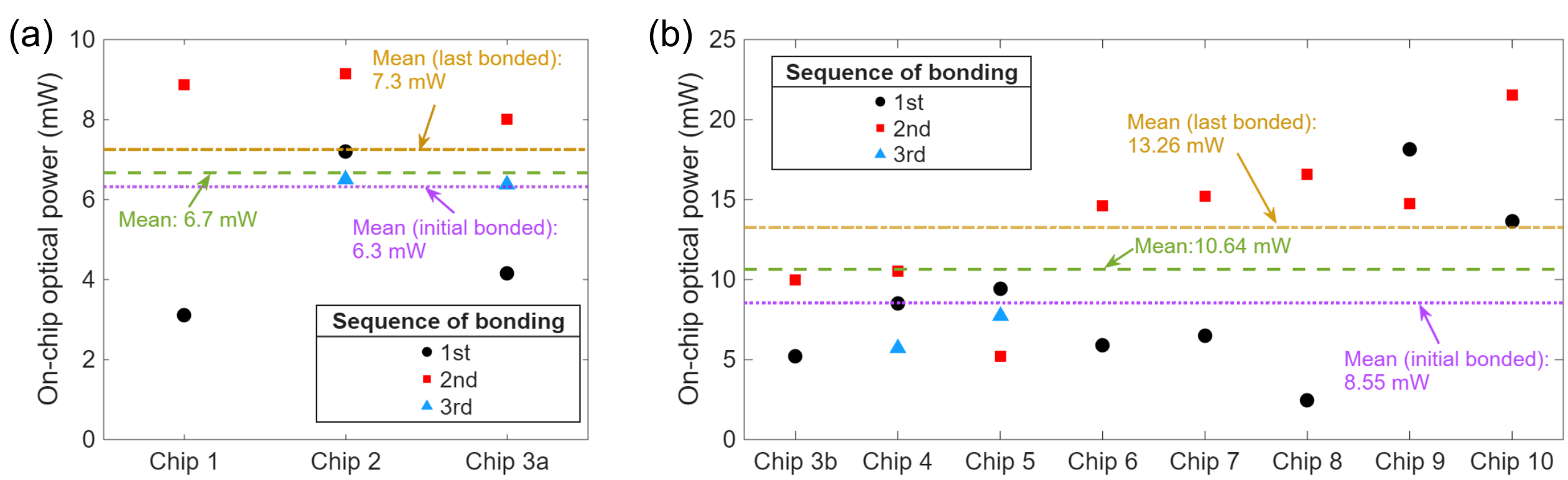

Figure S3: On-chip optical power of multiple LDs bonded on the same Si chip using (a) inverse taper and (b) taper couplers. The data correspond to a subset of samples from Figure 4(b). LD drive current: 50 mA. Legends denote the bonding sequence of LDs on each chip; measurements were taken after all LDs were bonded. Green dashed lines: mean on-chip optical power across all LDs per panel. Purple dashed lines: mean power of initial bonded samples (1st LD for sequences of two samples, 1st and 2nd for sequences of 3 LDs). Yellow dashed lines: mean power of last bonded samples (2nd or 3rd LD).

S3 Measurement of laser samples exhibiting thermal rollover

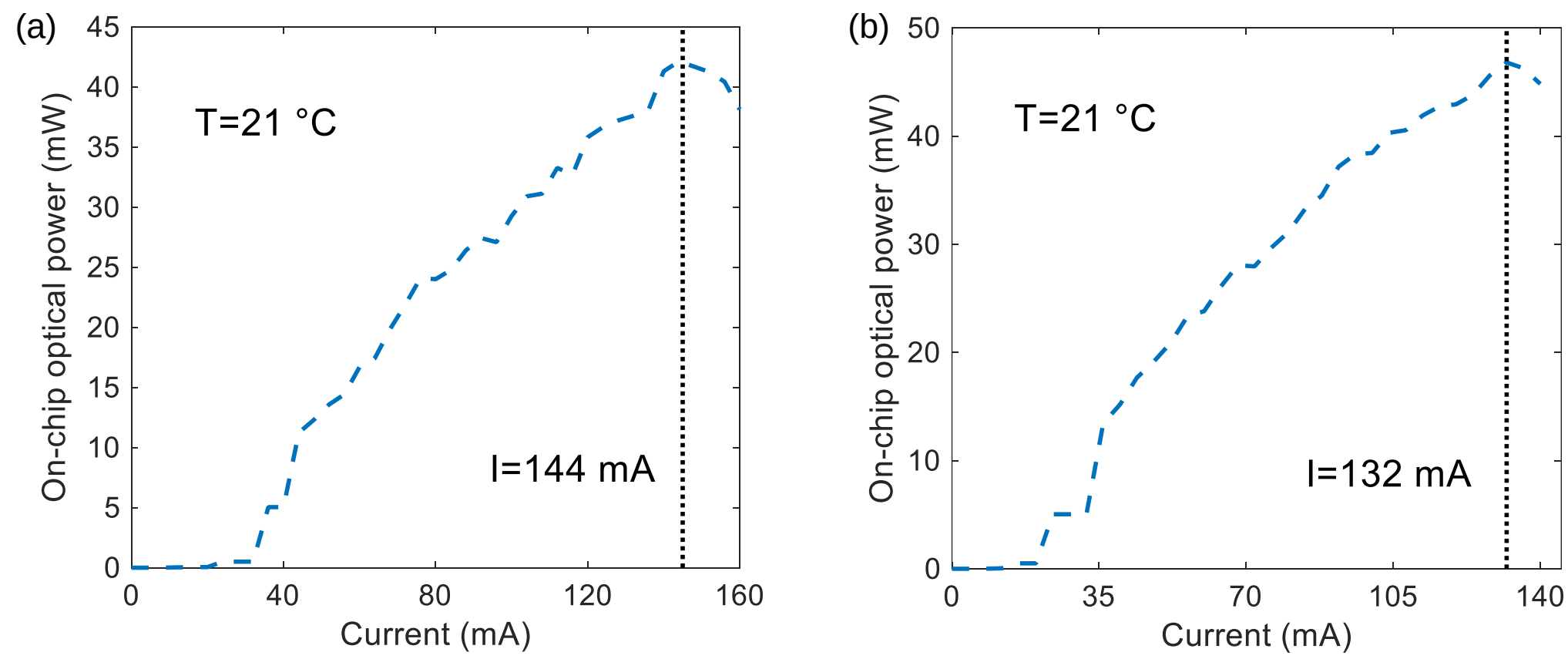

Figure S4: On-chip optical power (L) vs. current (I) curves of two representative flip-chip bonded laser diodes (LD1 and LD2). Thermal rollover was observed when the LD current exceeded 144 mA and 132 mA for (a) LD1 and (b) LD2, respectively.

The on-chip optical power vs. current (L-I) curves of two additional representative flip-chip bonded LD samples (LD1 and LD2) are shown in Figure S4(a) and Figure S4(b), respectively. Both samples used SiN taper designs for the LD-to-waveguide couplers. Thermal rollover was observed when the drive current exceeded 144 mA for LD1, with a peak on-chip optical power of 42.1 mW and corresponding on-chip wall-plug efficiency of ≈5.5%. LD2 exhibited thermal rollover at drive currents >132 mA, with a peak on-chip optical power of 46.8 mW and on-chip wall-plug efficiency of ≈7.8%. For both LD samples, the L-I curves were reproducible (under low drive currents without thermal rollover) before and after the onset of thermal rollover, indicating stable LD operation without observable LD degradation.

S4 Epoxy underfill and encapsulation of bonded lasers

We investigated a post-bonding epoxy underfill and encapsulation process to enhance the on-chip optical power of flip-chip-bonded LDs while improving mechanical robustness. Epoxy underfill was applied to five flip-chip-bonded LD samples to improve optical coupling to the SiN waveguide by reducing the large refractive index discontinuity at the LD–waveguide air gap. An optically transparent adhesive was first applied around the flip-chip-bonded LD using a thin metal rod applicator mounted on a micro-manipulator for precise control of the placement and volume. The bonded LDs were then encapsulated with thermally conductive epoxy to improve heat dissipation. Encapsulated devices are shown in Figure S5(a).

Various optically clear adhesives were evaluated for the underfill step. The UV-curable epoxies Delo OB6268 and Dymax 9622 caused loss of light transmission in the SiN waveguides when the LD current exceeded 45 mA (LD emission power 20 – 25 mW). This effect is likely due to degradation or burning of the adhesive under high-intensity blue light. Similar observations have been reported in our previous work [1]. To avoid this limitation, thermally-curable polydimethylsiloxane (PDMS, Sylgard 184) mixed at a 10:1 ratio (pre-polymer to cross-linker ratio) was chosen as the underfill epoxy. A thin metal rod (200 μm in diameter) was mounted on a micromanipulator (uMp-4, Sensapex) to enable precise PDMS application. The rod was first dipped into pre-mixed PDMS and then positioned near the laser diode to deposit a small amount of PDMS. The position of the rod was monitored using top- and side-view cameras. PDMS was thermally cured at 60 °C for 1 hour after application. With PDMS filling the LD-waveguide gap, the calculated Fresnel reflection at the coupling interface is estimated to decrease from ≈24.8% to ≈9.4% [2, 3].

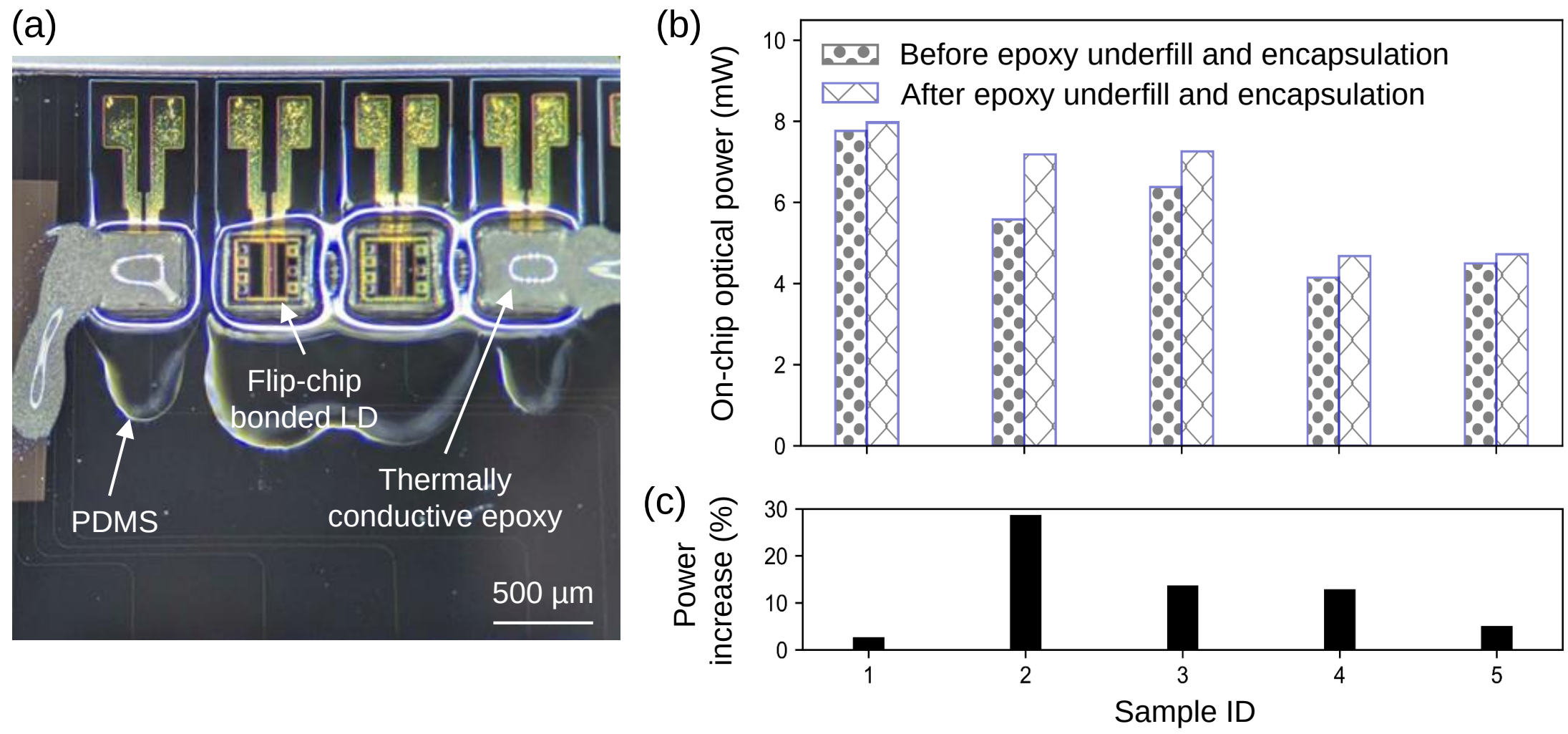

Figure S5: (a) Micrograph of flip-chip bonded LDs with underfill (with PDMS) and encapsulation (with thermally conductive epoxy). The two center LDs were underfilled, while the outer LDs underwent both underfill and encapsulation. (b) On-chip optical power comparison (at 50 mA LD current) before and after epoxy underfill and encapsulation. (c) Percent increase in optical power for the five LD samples after epoxy underfill and encapsulation.

After underfill with PDMS, the electrically insulating, thermally conductive epoxy, Duralco 128, was then applied over and around the LD to improve heat dissipation, followed by curing at 60 °C for 1 hour. A low curing temperature of 60 °C was chosen to avoid thermal damage to the LD while maintaining a moderate curing time. Both PDMS and Duralco 128 can also be cured at room temperature with an extended curing time. Different thermally conductive epoxies were evaluated following the PDMS underfill step. Among the tested epoxies (Duralco 128, Duralco 132, and Loctite Ablestik 2151), Duralco 128 showed the largest increase in optical output power, likely due to better thermal dissipation. The epoxy shown in Figure S5(a) is Duralco 132.

On-chip optical powers before and after PDMS underfill and Duralco 128 encapsulation are summarized in Figure S5(b). As shown in Figure S5(c), the on-chip optical power increased by ≈2.7% to ≈28.7% across the five samples after PDMS underfill and thermal epoxy encapsulation. The average power increase across the samples was 12.6%.

S5 Impact of the flip-chip process on laser diode characteristics

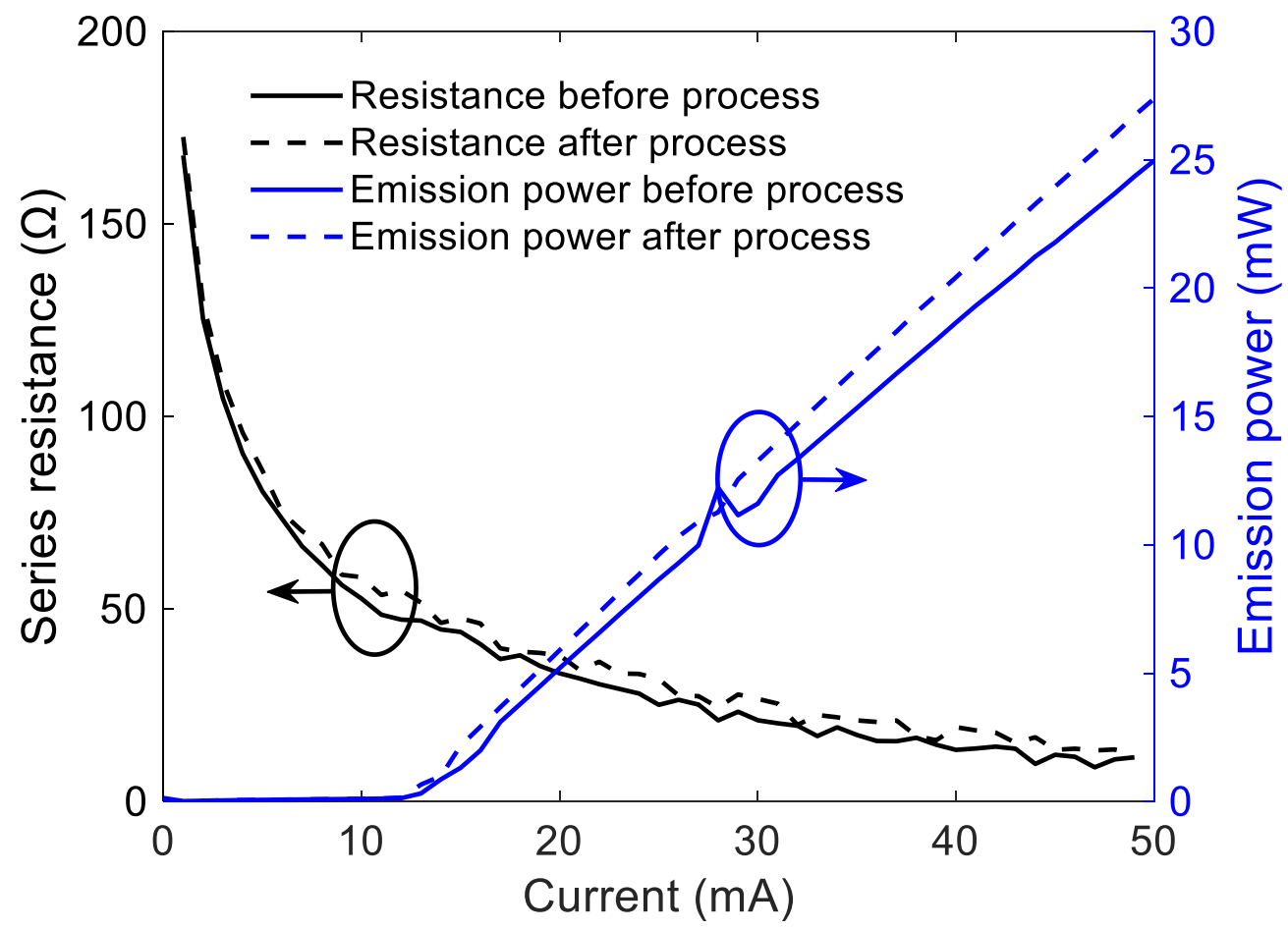

Figure S6: Series resistance and emission power of a representative LD before and after heat and pressure exposure.

Additional experiments were performed to investigate the impact of heat and pressure from the die bonder on LD performance. To directly measure LD emission power, independent of alignment-sensitive waveguide coupling, the LDs were mounted face-up on a Si dummy chip without solder. This configuration limited testing to an inverted arrangement, where the pickup tool applied heat and pressure to the LD top (contact) surface rather than the bottom surface (as in the flip-chip bonding procedure). Series resistance and emission power were measured before and after exposure.

First, to measure the LD characteristics prior to heat and pressure application (exposure process), three LDs were positioned with metal contacts facing up near the facet of a dummy Si chip (identical dimensions as the Si photonic chips used in the flip-chip bonding process). A thin layer of uncured metal adhesive (Loctite, Ablebond 84-1LMIT1) secured the LDs to the Si chip. Without curing of the metal adhesive, L-I-V curves before the exposure process were collected with a source measure unit (SMU) and a power meter, with DC needles probing the P and N contacts of the LDs. The LD emissions were directly incident on a free-space detector for emission power measurements. Next, the LDs on the Si dummy chip were mounted in the die bonder, and the pickup tool touched down onto each LD, following the same heating and force profile as the flip-chip bonding process. As in that process, heat from the die bonder stage was also applied. L-I-V curves of the LDs were then measured using the same procedure as prior to the exposure process.

Figure S6 compares the series resistance and emission power of a representative LD sample before and after heat and pressure exposure from the die bonder, with the resistance extracted from the measured I–V curve. After the process, the series resistance increased slightly, by ≈14.7% (3.8 Ω) on average compared to its initial values. The emission power at 50-mA drive current increased slightly (≈9.7%) after the process, which may be attributed to thermal curing of the metal adhesive. The applied pressure during the exposure process likely pressed the LD and dummy Si chip into closer contact, which may have altered the thermal conduction properties through the cured metal adhesive. The other two LD samples showed similar results of increased series resistance (≈14.4% and ≈11.6% increase on average) and emission power (≈12.8% and ≈17.3% increase on average). Overall, the results imply that changes in LD performance during the flip-chip bonding process are likely to be small. A limitation of these tests is the manner in which force was applied. Here, force was exerted on the top surface of each LD while the bottom surface contacted the Si dummy chip. By contrast, during flip-chip bonding, force was applied to the back of the LD with its stoppers contacting the Si photonic chip.

S6 Schematic of the measurement setup for on-chip power monitoring

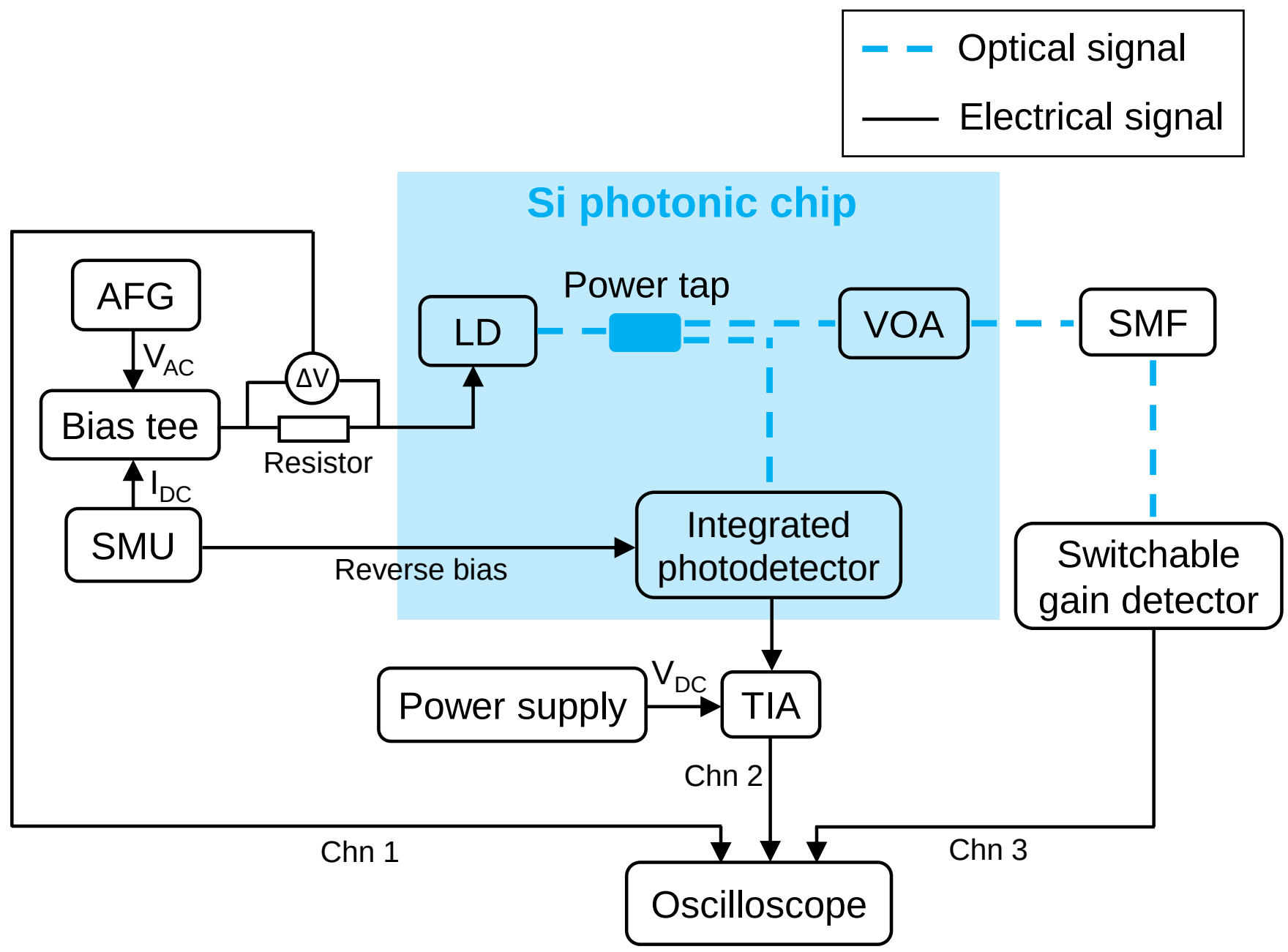

Figure S7: Schematic of the measurement setup for power monitoring with the photonic integrated circuit (Figure 8). AFG: arbitrary function generator, SMU: source measure unit, VOA: variable optical attenuator, TIA: transimpedance amplifier, SMF: single-mode fiber, Chn: oscilloscope channel.

Figure S7 illustrates the experimental setup used for the on-chip power monitoring demonstration in Figure 8. The photonic integrated circuit (PIC, on the Si photonic chip) consisted of a flip-chip bonded LD, an optical power tap, an integrated photodetector used as a power monitor, and a thermo-optic switch used as a variable optical attenuator (VOA). A two-channel source measure unit delivered a DC current bias to the LD and a reverse bias voltage to the integrated photodetector. The LD was directly modulated with a sine-wave AC signal, generated by an arbitrary function generator (AFG). A 10-Ω sensing resistor was connected in series with the LD to monitor the current change during LD modulation. A bias tee combined the DC and AC LD drive signals. Light coupled into the on-chip SiN waveguide from the bonded LD was split into two paths via the power tap — the tap path directed a fraction of the light to the integrated photodetector, and the thru path routed light through the VOA to an output fiber-to-chip edge coupler, which was coupled to a single-mode fiber (SMF). The photocurrent of the integrated photodetector was amplified by a transimpedance amplifier (TIA). The PIC output was routed to an off-chip switchable-gain detector through the SMF. The voltage drop across the resistor was measured with an oscilloscope for subsequent current calculations (Chn 1). The amplified photocurrent signal (Chn 2) and output power signal (Chn 3) were simultaneously collected by the oscilloscope. The oscilloscope was internally triggered by the voltage drop across the resistor at Chn 1, ensuring synchronized signal acquisition across the 3 channels.

S7 Quantifying dynamic optical power during LD modulation

The dynamic optical power during sinusoidal laser modulation (reported in Section 5.2 and Figure 8) was calculated from the measured oscilloscope traces using the following analysis and procedure.

In the DC LD driving case, the relationship between the output optical power from the PIC incident on the off-chip switchable-gain detector (P_0) and the measured oscilloscope voltage magnitude (V_0) can be described by Equation 1:

$$V_0 = GRP_0 + F, \tag{1}$$

where G and R are the gain and responsivity of the off-chip detector, and F is the DC voltage offset of the oscilloscope trace with no optical power at the detector. Equation 1 can be further simplified as:

$$V_0 = aP_0 + b, \tag{2}$$

where P_0 and V_0 follow a linear relationship, a and b being the slope and intercept of the linear function.

During sinusoidal modulation of the LD drive current, the measured time-dependent oscilloscope voltage magnitude, $V(t)$, can be further described by Equation 3 (neglecting phase delay between V and P):

$$V(t) = V_0 + \Delta V \sin(2\pi f t) = a[P_0 + |H(f)|\Delta P \sin(2\pi f t)] + b, \tag{3}$$

where ΔV and ΔP are the amplitude of the voltage signal and the corresponding off-chip optical power modulation. $|H(f)|$ is the attenuation factor determined by the 3-dB optoelectronic bandwidth, BW, of the detector, and f is the sinusoidal modulation frequency. The dependence of the attenuation factor (represented by the frequency response) on the detector's bandwidth is approximated in Equation 4 as follows:

$$|H(f)| = \frac{1}{\sqrt{1 + (f/BW)^2}}. \tag{4}$$

Comparing Equations 2 and 3, the amplitude of the dynamic off-chip optical power during modulation, ΔP, can be derived from Equation 5:

$$\Delta P = \frac{\Delta V}{a|H(f)|}, \tag{5}$$

and the maximum and minimum dynamic off-chip optical power during LD modulation can be calculated as $P_{max} = P_0 + \Delta P$ and $P_{min} = P_0 - \Delta P$, where P_0 represents the mean of the dynamic optical power.

To determine the parameters a and b, V_0 (at oscilloscope Chn3, Figure S7) was recorded for a series of DC LD drive currents, while P_0 was measured at each of these drive currents using a power meter. Following Equation 2, a linear fit was performed to obtain a and b. $|H(f)|$ at $f = 1$ MHz was then calculated from the specified optoelectronic bandwidth of the detector ($BW = 1.6$ MHz in this work) using Equation 4. Finally, with a, $|H(1MHz)|$, and b, Equation 3 was applied to compute P_{max}, P_0, and P_{min} from $V(t)$ during 1 MHz sinusoidal modulation of the LD drive current.

References

[1] D. A. Roszko, *et al.*, "Foundry-fabricated dual-color nanophotonic neural probes for photostimulation and electrophysiological recording," *Neurophotonics*, vol. 12, no. 2, pp. 025 002–025 002, 2025.

[2] X. Li, *et al.*, "Improved multiple-wavelength Brillouin–Raman fiber laser assisted by four-wave mixing with a micro-air cavity," *Appl. Opt.*, vol. 54, no. 33, pp. 9919–9924, 2015.

[3] Z. Cai, W. Qiu, G. Shao, and W. Wang, "A new fabrication method for all-PDMS waveguides," *Sens. Actuators A: Phys*, vol. 204, pp. 44–47, 2013.
